# Comparison of the Effect of Daily Vitamin D2 and Vitamin D3 Supplementation on Serum 25-Hydroxyvitamin D Concentration (Total 25(OH)D, 25(OH)D2, and 25(OH)D3) and Importance of Body Mass Index: A Systematic Review and Meta-Analysis

**DOI:** 10.1016/j.advnut.2023.09.016

**Published:** 2023-10-20

**Authors:** Ellen GHM. van den Heuvel, Paul Lips, Linda J. Schoonmade, Susan A. Lanham-New, Natasja M. van Schoor

**Affiliations:** 1 Scelta Mushrooms B.V., Venlo, The Netherlands; 2 Amsterdam UMC location Vrije Universiteit Amsterdam, Department of Internal Medicine, Endocrine section, Amsterdam, The Netherlands; 3 Vrije Universiteit Amsterdam, Medical Library, Amsterdam, The Netherlands; 4 Department of Nutrition, Food & Exercise Sciences, University of Surrey, Faculty of Health & Medical Sciences, School of Biosciences, United Kingdom; 5 Department of Epidemiology and Data Science, Amsterdam UMC location Vrije Universiteit Amsterdam, Amsterdam, The Netherlands; 6 Amsterdam Public Health Research Institute, Aging & Later Life, Amsterdam, The Netherlands

**Keywords:** healthy adults, systematic review, meta-analysis, ergocalciferol, cholecalciferol, bioavailability, 25(OH)D, vitamin D response

## Abstract

**Background:**

Two previous meta-analyses showed smaller differences between vitamin D3 and vitamin D2 in raising serum 25-hydroxyvitamin D [25(OH)D] and a consistently high heterogeneity when only including daily dosing studies.

**Objective:**

This study aimed to compare more frequently dosed vitamin D2 and vitamin D3 in improving total 25(OH)D and determine the concomitant effect of response modifiers on heterogeneity, and secondly, to compare the vitamin D2-associated change in 25(OH)D2 with the vitamin D3-associated change in 25(OH)D3.

**Methods:**

PubMed, EMBASE, Cochrane, and the Web of Science Core collection were searched for randomized controlled trials of vitamin D2 compared with vitamin D3, daily or once/twice weekly dosed. After screening for eligibility, relevant data were extracted for meta-analyses to determine the standardized mean difference when different methods of 25(OH)D analyses were used. Otherwise, the weighted mean difference (WMD) was determined.

**Results:**

Overall, the results based on 20 comparative studies showed vitamin D3 to be superior to vitamin D2 in raising total 25(OH)D concentrations, but vitamin D2 and vitamin D3 had a similar positive impact on their corresponding 25(OH)D hydroxylated forms. The WMD in change in total 25(OH)D based on 12 daily dosed vitamin D2-vitamin D3 comparisons, analyzed using liquid chromatography-tandem mass spectrometry, was 10.39 nmol/L (40%) lower for the vitamin D2 group compared with the vitamin D3 group (95% confidence interval: −14.62, −6.16; *I*^*2*^ = 64%; *P* < 00001). Body mass index (BMI) appeared to be the strongest response modifier, reducing heterogeneity to 0% in both subgroups. The vitamin D2- and vitamin D3-induced change in total 25(OH)D lost significance predominantly in subjects with a BMI >25 kg/m^2^ (*P* = 0.99). However, information on BMI was only available in 13/17 daily dosed comparisons.

**Conclusions:**

Vitamin D3 leads to a greater increase of 25(OH)D than vitamin D2, even if limited to daily dose studies, but vitamin D2 and vitamin D3 had similar positive impacts on their corresponding 25(OH)D hydroxylated forms. Next to baseline 25(OH)D concentration, BMI should be considered when comparing the effect of daily vitamin D2 and vitamin D3 supplementation on total 25(OH)D concentration.

This study was registered in PROSPERO as CRD42021272674.

**Statement of significance**

Previous meta-analyses suggest that vitamin D3 may be more potent in increasing serum 25-hydroxyvitamin D [25(OH)D] concentrations than vitamin D2. In addition, it appears that with daily dosing, this difference is smaller compared with other doses, e.g., monthly/bolus. Our meta-analysis confirms this when comparing the commonly recommended more frequent dosing regimens, daily compared with weekly, although residual heterogeneity remained high. Body mass index and baseline 25(OH)D concentration may contribute to this residual variability and may therefore be considered when recommending a daily intervention with vitamin D2 or vitamin D3.

## Introduction

Vitamin D is available in 2 distinct forms, namely, ergocalciferol or vitamin D2 and cholecalciferol or vitamin D3. The naturally occurring plant-derived form, vitamin D2, was produced in the early 1920s through UV exposure of foods, such as yeast and mushrooms [1]. Vitamin D3 is synthesized in the skin of humans from 7-dehydrocholesterol and is also present in animal-based foods, such as egg yolks and oily fish. Both vitamin D3 and vitamin D2 are synthesized commercially and found in dietary supplements or fortified foods [2]. Although much of the vitamin D in the diet is in the form of vitamin D3, vitamin D2 may be an underestimated contributor to the total 25-hydroxyvitamin D [25(OH)D] as 25(OH)D2 was detected in 79% of the sera of Irish adults [3]. Two meta-analyses indicated that vitamin D3 is more potent in raising serum 25(OH)D concentrations than vitamin D2. The difference in vitamin D2 and vitamin D3 efficacy was lower when restricted to studies with a daily dosing regimen than studies with dosing regimens other than daily, such as bolus (*P* < 0.0001) [4] and monthly dosing (*P* = 0.16) [5]. However, residual heterogeneity remained high. It is not clear which factors contributed to this residual heterogeneity, providing valuable information for better targeting and application of the daily intervention, which would be useful for public health and practice. Confounding factors may be baseline vitamin D status, but also BMI, as both were found to be associated with response to vitamin D supplementation [6]. However, the effect of these factors on the response may be different for vitamin D2 and vitamin D3. Often daily or weekly administration of cholecalciferol is recommended. Thus far, no meta-analyses or studies have compared the efficacy of vitamin D2 and vitamin D3 taking into account the more frequent dosing regimens only, e.g., daily compared with once or twice a week.

In addition, no meta-analysis compared the vitamin D2-induced change in 25(OH)D2 with the vitamin D3-induced change in 25(OH)D3. A significant negative association between baseline total 25(OH)D concentration (i.e., serum 25(OH)D2 plus serum 25(OH)D3) and response to vitamin D2 or vitamin D3 treatment has been found in a number of studies [[7], [8], [9]]. The impact of baseline total 25(OH)D concentrations might be different for vitamin D2 and vitamin D3, as serum 25(OH)D2 represents only 7% of the total serum 25(OH)D concentration [3]. A previous meta-analysis showed that when the baseline concentration of 25(OH)D was high, consisting mostly of 25(OH)D3, consumption of UV-exposed mushrooms containing vitamin D2 does not lead to a higher serum total 25(OH)D concentrations. This seemed to be due to a reduction in serum 25(OH)D3 concentrations that accompanied the increase in 25(OH)D2 following D2 supplementation [10]. An analogous phenomenon to a similar extent occurred with vitamin D3 supplementation after increasing baseline concentrations of 25(OH)D2: 25(OH)D3 supplementation increased 25(OH)D3 and decreased 25(OH)D2 [11]. However, when there is a high total 25(OH)D concentration at baseline, it usually consists mainly of 25(OH)D3 because, unlike serum 25(OH)D2, 25(OH)D3 is directly influenced by skin exposure to UVB from sunlight [12]. High serum 25(OH)D3 concentration may reduce the vitamin D2-induced increase in total 25(OH)D. To minimize this impact, the vitamin D2-induced change in 25(OH)D2 should be compared with the vitamin D3-induced change in 25(OH)D3.

The aim of this current meta-analysis was 3-fold: *1*) to compare vitamin D2 and vitamin D3 in improving total 25(OH)D in those healthy adult randomized controlled trials (RCTs) in which vitamin D was more frequently administered, e.g., daily compared with once or twice a week; *2*) to compare vitamin D2-associated change in 25(OH)D2 and vitamin D3-associated change in 25(OH)D3; and *3*) to determine the concomitant effect of BMI, baseline vitamin D status, and other response modifiers on the effectiveness of daily dosed vitamin D2 and vitamin D3 in raising total 25(OH)D.

## Methods

This systematic review was carried out in accordance with the PRISMA statement [13]. Registration on PROSPERO (CRD42021272674) can be found at https://www.crd.york.ac.uk/prospero/display_record.php?RecordID=272674. A comprehensive search was performed in the bibliographic databases PubMed, Embase.com, the Cochrane Library (via Wiley), and the Web of Science Core collection from inception to 7 June 2022, in collaboration with a Medical Librarian (LS). Search terms included controlled terms (MeSH in PubMed and Emtree in Embase) as well as free-text terms. The following terms were used (including synonyms and closely related words) as index terms or free-text words: “*ergocalciferol or vitamin D2*” and “*cholecalciferol or vitamin D3.”* The search was performed without date or language restrictions. A search filter was applied to limit to randomized controlled trials. The Cochrane Library search also included vitamin D status or 25(OH)D. Duplicate articles were excluded by LS using Endnote X20.0.1 (Clarivate), following the Amsterdam Efficient Deduplication method [14] and the Bramer-method [15]. The full search strategies for all databases can be found in Supplementary Table S1.

### Selection process

Two reviewers (EvdH and NMvS) independently screened all potentially relevant titles and abstracts for eligibility using Rayyan [16]. Studies were included if they met the following criteria: *1*) randomized controlled trials; *2*) healthy adults aged >18 y of any sex and race; *3*) the intervention contained a comparison between vitamin D2 and vitamin D3; and *4*) effective outcome data was change in total 25(OH)D, 25(OH)D2, and/or 25(OH)D3 over time. Studies were excluded for the following reasons: *1*) review or background article; *2*) different population than defined in the inclusion criteria; *3*) nonrandomized trial; *4*) protocol; *5*) treatment that fails to inclusion criteria, i.e., no comparable dose or dosing regimen for vitamin D2 and vitamin D3 or vitamin D combined with other therapies (e.g., medication, nutrients except for calcium); *6*) other dosing regimens than daily or once or more times a week (e.g., single dose, twice weekly, monthly); or *7*) outcome other than 25(OH)D or its isomers. If necessary, the full text article was checked for eligibility criteria.

### Data extraction

EvdH extracted and PL verified *1*) sample size; *2*) baseline 25(OH)D concentration; *3*) results; and *4*) method of measurement of 25(OH)D. For the results, quantitative data on average change and SD of the change in total 25(OH)D, 25(OH)D2, and/or 25(OH)D3 from baseline were extracted to calculate effect size. In case the studies reported only baseline and final concentrations, the mean and SD of the change was computed using the formula ${\text{SD}}_{E,\text{change}}=\sqrt{{\text{SD}}_{E,\text{baseline}}^{2}+{\text{SD}}_{E,\text{final}}^{2}-\left(2\times \text{Corr}\times {\text{SD}}_{E,\text{baseline}}\times {\text{SD}}_{E,\text{final}}\right)}$ with a correlation coefficient of 0.8. The SD was derived from confidence interval (CI) using the formula SE=(upperlimit−lowerlimit)/3.92 [17].

EvdH extracted and SALN verified the rest of the data using a standard data extraction form. This included the following: *1*) general information (e.g., the first author’s name, the publication year, latitude at which the study was performed); *2*) subject characteristics (e.g., sex, age, race, percentage of subjects with serum concentration <50 nmol/L 25(OH)D at baseline, BMI, and compliance); and *3*) interventions (vitamin D dose and whether this dose was reanalyzed, carrier of vitamin D, dosing regimen, duration, and whether calcium intake was same for both treatments). In addition, EvdH extracted and PL and NMvS verified the methodological quality of the full text papers [18]. When high risk of bias for one or more key domains was found, the study was classified as being of “high risk” of bias [18]. Differences in judgment were resolved through a consensus procedure.

### Potential factors explaining heterogeneity

Although only studies with more frequent dosing were included, dosing frequency may affect the outcome [4,5]. Therefore, the meta-analysis was stratified on frequency of supplementation (daily compared with one or more times a week). Further limiting to RCTs that daily dosed vitamin D, a number of subgroup analyses were performed to examine potential effects of response modifiers on heterogeneity, i.e., <50 nmol/L 25(OH)D at baseline [19,20], subject characteristics such as sex and BMI, latitude of study location, dose of vitamin D, and presence of calcium. Justification for these choices of factors include the fact that women have been reported to have a greater 25(OH)D response to vitamin D2 than men [9]; low serum 25(OH)D concentration has been reported in older adults with overweight or obesity [21,22] because baseline 25(OH)D concentration has an impact on the efficacy of vitamin D to increase serum 25(OH)D [19,20], BMI may interfere with the outcome. Another moderator may be the latitude of study location; a greater and significant increase in serum total 25(OH)D with consumption of UV-exposed mushrooms was found at >45°N compared with <45°N [10]. In addition, calcium intake may interfere. A negative association between calcium intake and serum 25(OH)D was found, at least in subjects with an adequate vitamin D intake [23]. Therefore, the RCTs with a daily dosing regimen were stratified on the following: *1*) described percentage of subjects with baseline 25(OH)D concentration of <50 nmol/L, ≤60% or >60%; *2*) subject characteristics, such as race with >50% Caucasians or ≤50%, age with <65 y or ≥65 y, sex with >70% women or ≤70%, and average BMI with cutoff value of 25 kg/m^2^; *3*) latitude at which the study was conducted with <30°N, 30 to <45°N or >45°N; *4*) average daily dose of ≤25 μg or >25 μg as lower dosage may result in smaller differences in efficacy [5]; and *5*) coadministration of calcium in the vitamin D2 and vitamin D3 treatments (yes/no).

### Statistical analysis and sensitivity analyses

The meta-analysis to compare the efficacy of vitamin D2 with that of vitamin D3 in improving vitamin D status was carried out with Review Manager version 5.2 (Cochrane Collaboration) with random-effects analysis to determine the standardized mean difference (SMD) because different methods of analyses were used. When studies were included that analyzed serum 25(OH)D using liquid chromatography-tandem mass spectrometry (LC-MS/MS), the overall weighted mean difference (WMD) was determined.

Sensitivity analyses were performed both on all studies independent of dosing regimen as well as limited to studies with a daily dosing regimen, by *1*) including only intention to treat (ITT) or per protocol (PP) analyses, or by excluding data from *2*) studies with “high risk” of bias (see Supplementary Table S3); or *3*) studies in which the total 25(OH)D was based on the measurement of 25(OH)D2 and 25(OH)D3 by LC-MS/MS. In addition to forest plots, the presence of statistical heterogeneity (*I*^*2*^) was examined using the χ^2^ statistic. An *I*^*2*^ of 0%–40% might not be important, whereas 30%–60% may represent moderate heterogeneity, 50%–90% substantial heterogeneity, and 75%–100% considerable heterogeneity [17].

Evidence of publication bias was assessed using funnel plots in addition to searching for unpublished studies through the Cochrane database. Two-sided *P* value of <0.10 was considered statistically significant for the subgroup analysis [24].

## Results

The literature search generated a total of 1797 references: 352 in PubMed, 691 in Embase, 226 in Web of Science, and 528 in the Cochrane Library. After removing duplicates of references that were selected from more than one database, 1351 references remained. The flowchart of the search and selection processes is presented in Supplementary Figure S2. Our screening yielded 17 studies with 20 comparisons between vitamin D2 with vitamin D3, of which 3 included vitamin D2-vitamin D3 comparisons maintaining a weekly dosing regimen [[25], [26], [27]]. In the weekly dosing study of Nasim et al. [27], subjects were excluded when 25(OH)D concentrations exceeded 75 nmol/L after 8 wk, therefore only the 8-wk results are included in the current meta-analysis. All the RCTs provided extractable data on serum total 25(OH)D concentration, whereas extractable data on 25(OH)D2 and 25(OH)D3 concentrations were present for 9 vitamin D2-vitamin D3 comparisons.

**FIGURE 1:**
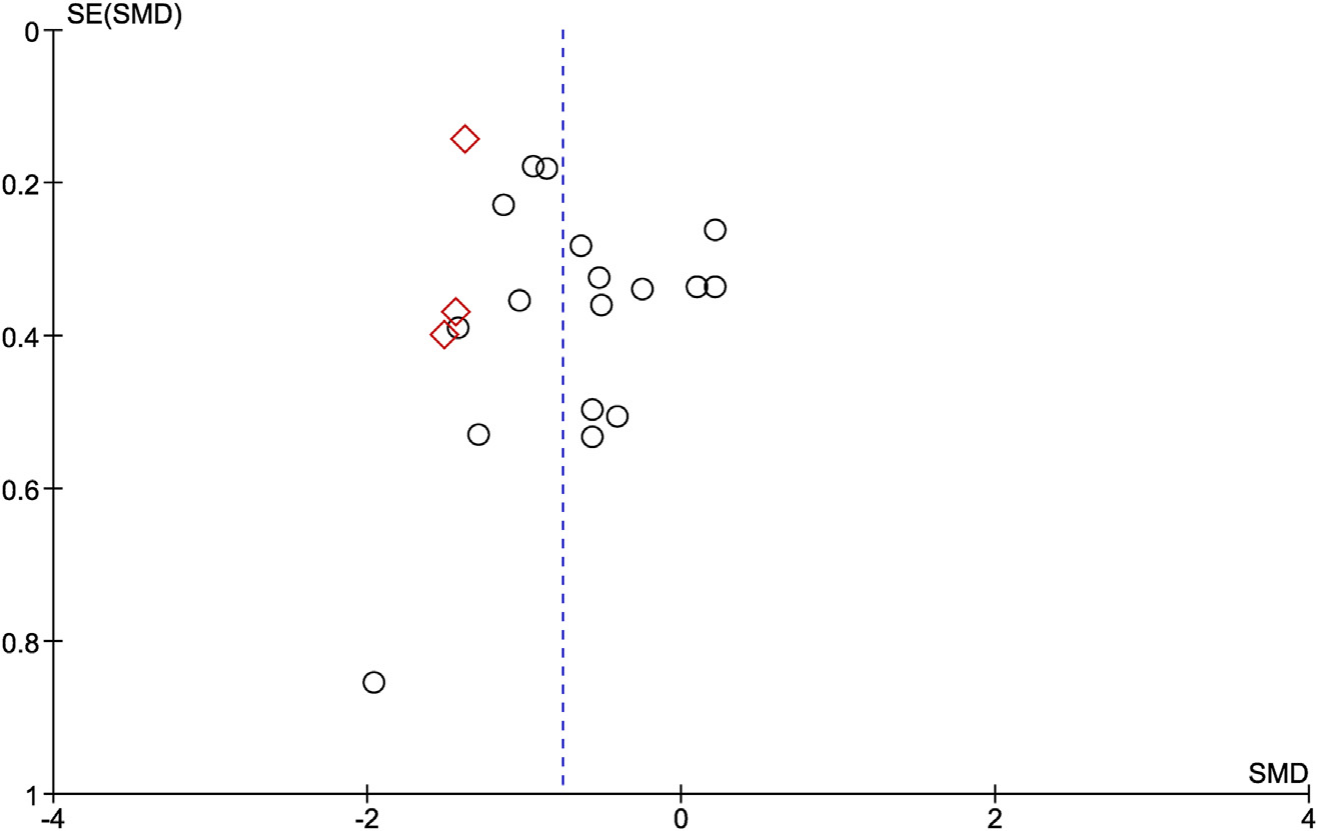
Funnel plot of all included studies comparing vitamin D2 and D2 in changing serum concentration of total 25(OH)D. ◊, weekly treatment; ○, daily treatment.

Limited to the 17 vitamin D2-vitamin D3 comparisons based on a daily dosing regimen [7,9,[28], [29], [30], [31], [32], [33], [34], [35], [36], [37], [38], [39]], one study was conducted in patients with posthip fracture [28], whereas the others were performed in healthy adults. Basic health checks were not described in 2 studies [7,32]. The other studies considered different diseases and medications that can interfere with vitamin D metabolism and sometimes the concentration of different blood [9,27,28,33,36] and urine markers [26,29]. Except for 2 studies, 1 on BMI [37] and 1 on serum values [35], none of the studies used the outcomes of these basic health checks in the statistics. Three studies were conducted in women [35,37,38]; 1 study did not provide the sex of the subjects [28], and the other 13 vitamin D2-vitamin D3 comparisons were studied in men and women. The follow-up duration of the studies varied between 4 and 48 wk. Two studies did not verify vitamin D content of the supplementation properly [28,38,39], and in the study of Glendenning et al. [28], this analysis was performed by each individual supplier of the vitamin D supplement. In 4 of the verifying analyses, the vitamin D content of the supplementation appeared to differ by >10% of the target treatment dose between treatment groups [33,34,36]. In some studies calcium was included in the vitamin D2 or vitamin D3 supplements [28,38,39]. More details of the included studies are shown in Table 1, TABLE 2. The funnel plot shown in Figure 1 includes all daily and weekly dosing studies and for the studies present, and there were no signs of asymmetry in terms of effect size being positive or negative. However, there were very few studies toward the base of the funnel, which could possibly suggest publication bias against smaller studies.

**Table 1:** Subject characteristics

Ref	Country (latitude)	Healthy (male %)	Age range (y)		Race^1^	Baseline 25(OH)D (nmol/L)				% participants with baseline <50 nM 25(OH)D		% BMI ≤25^2^	Compliance (%)
			low	high		D2 group		D3 group		D2	D3		
Hartwell [38]	Denmark (56N)	Healthy (0)	22	49	100	74.2	15.3	77.5	15.6	ND		ND	PP: ND
Trang [7]	Canada (44N)	Healthy (63)	36	40	33	43.7	17.7	41.3	17.7	56	69	ND	PP: ND
Holick [30]	United States (42N)	Healthy (31)	18	81	28	42.3	26.3	49.0	27.8	60		*ND; mean BMI=31*	PP=ITT: D2 94; D3 95
Glendenning [28]	Australia (31S)	Hospitalized (ND)	82	84	ND	PP: 39.2 ITT: 37.2	12.2 14.4	43.3 42.4	22.3 27.9	100		ND	PP: ≥80%. ITT: D2 59; D3 47 (NS)
Binkley [29]	United States (43N)	Healthy (36)	65	88	95	80.0	21.0	74.8	25.0	ND		0	PP=ITT: D2 95; D3 92
Heaney [25]	USA (42N)	Healthy (9)	46	52	100	76.5	37.0	65.0	23.0	ND		D2 45; D3 37	PP: 100
Lehman [33]	Germany (51 N)	Healthy (35)	30	40	ND	37.6	13.3	43.7	23.3	85	69	D2 74; D3 71	PP: 97
Nimitphong [39]	Thailand (14N)	Healthy (18)	34	39	0	51.8	16.6	53.2	16.1	53	70	100	PP: 90
Logan [34]	New Zealand (46S)	Healthy (21)	18	50	84	PP: 74.0 ITT: 69.0	20.2 23.0	80.0 79.0	12.2 14.0	5		100	PP: ≥90%. ITT: ND
Keegan [32]	United States (42N)	Healthy (24)	*Mean age: 35*		ND	48,5	16.3	42.8	6.1	ND		ND	PP: ND
Itkonen [35]	Finland (60N)	Healthy (0)	20	37	100	63.5	11.3	66.6	14.8	11	0	D2 89; D3 75	PP: 97 (NS)
Shieh [26]	United States (34N)	Healthy (ND)	45	62	58	55.5	8.3	58.3	18.0	26	21	D2 46; D3 51	PP: ≥80
Hammami [9]	Saudi Arabia (25N)	Healthy (44)	31	38	0	39.5	12.2	41.3	10.7	100 (at enrollment)		D2 56; D3 41	PP: D2 98; D3 99
Nasim [27]	Dubai (25N)	Healthy (48)	46	52	0	ND		ND		ND	ND	ND	PP: 100
Biancuzzo-S [31]	United States (42N)	Healthy (39)	18	81	28	41.5	24.8	49.0	27.8	64		*ND; mean BMI=30*	PP: D2 94; D3 95
Biancuzzo-J [31]	United States (42N)	Healthy (31)	19	73	23	39.5	25.0	44.8	27.8	64		*ND; mean BMI=29*	PP: D2 94; D3 95
Fisk-5 [36]	United Kingdom (52N)	Healthy (38)	21	38	75	48.0	26.6	31.3	22.1	38	86	D2 100; D3 57	PP: 100
Fisk-10 [36]	United Kingdom (52N)	Healthy (50)	22	38	81	41.9	14.1	30.9	29.1	63	75	D2 75; D3 63	PP=ITT: 100
Tripkovic-J [37]	United Kingdom (51N)	Healthy (0)	40	47	80	ITT: 44.9	29.7	42.3	29.5	ND		D2 59; D3 63	ITT: 94
Tripkovic-B [37]	United Kingdom (51N)	Healthy (0)	40	47	78	ITT: 46.1	30.1	41.9	29.2	ND		D2 58; D3 62	ITT: 94

Abbreviations: BMI, body mass index; ITT, intention to treat; ND, not described; NS, described as not significantly different; PP, per protocol.

1 Percentage of Caucasian subjects.

2 Percentage of subjects with BMI ≤ 25 kg/m^2^.

**TABLE 2:** General information on intervention and quality of studies

Ref	Dose (μg)	D2/D3 content reanalyzed? (%)^1^	Carrier vitamin D	Dosing regimen	Duration (wk)	Ca (mg/d)^2^	Method of analyses^3^	ITT or PP data available	Quality^4^
Hartwell [38]	100	no	Supplement	daily	8	yes (500)	UV absorption^c^	PP	UC
Trang [7]	100	yes (ND)	Supplement	daily	2	no	radio-immune assay^a^	PP	UC
Holick [30]	25	yes (<10)	Supplement	daily	11	no	HPLC-MS/MS^c^	ITT & PP^5^	UC
Glendenning [28]	25	no	Supplement	daily	12	yes (240)	HPLC^b^	ITT & PP^6^	UC
Binkley [29]	40	yes (D2 +7; D3 +4)	Supplement	daily	48	no	HPLC^a^	ITT & PP^5^	UC
Heaney [25]	179	yes (D2 −6; D3 +11)	Supplement	weekly	12	no	Chemiluminescent assay, DiaSorin^d^	PP	H
Lehman [33]	50	yes (D2 −4; D3 +8)	Supplement	daily	8	no	HPLC-MS/MS^d^	PP	H
Nimitphong [39]	10	no	Supplement	daily	12	yes (D2 1000; D3 675)	HPLC-MS/MS^b^	PP	H
Logan [34]	25	yes (D2 +28; D3 +12)	Supplement	daily	25	no	HPLC-MS/MS^c^	PP&ITT	H
Keegan [32]	50	yes (<10)	Supplement	daily	12	no	HPLC-MS/MS^d^	PP	H
Itkonen [35]	25	yes (D2 −2; D3 0)	Supplement	daily	8	no	HPLC-MS/MS^a^	PP	L
Shieh [26]	357	yes (ND)	Supplement	twice a week	5	no	Chemiluminescent assay, DiaSorin^b^	PP	UC
Hammami [9]	45	yes (D2 −8; D3 −11)	Supplement	daily	20	no	HPLC^c^	PP	L
Nasim [27]	179	no	Supplement	weekly	8	no	Electro-chemiluminescence^c^	PP	H
Biancuzzo-S [31]	25	yes (<10)	Supplement	daily	11	no	LC-MS/MS^c^	PP	UC
Biancuzzo-J [31]	25	yes (<10)	Orange juice	daily	11	no	Idem	PP	UC
Fisk-5 [36]	5	yes (D2 −4; D3 +4)	Malted milk drink	daily	4	no	LC-MS/MS^a^	PP	H
Fisk-10 [36]	10	yes (D2 −25; D3 0)	Malted milk drink	daily	4	no	Idem	ITT & PP^5^	H
Tripkovic-J [37]	15	yes (<10)	Orange juice	daily	12	no	LC-MS/MS^a^	ITT	L
Tripkovic-B [37]	15	yes (<10)	Biscuit	daily	12	no	Idem	ITT	L

Abbreviations: CV, coefficient of variation; DEQAS,; HPLC, high-performance liquid chromatography; ITT, intention to treat; LC, liquid chromatography; MS, mass spectrometry; PP, per protocol.

1 Percentage deviation from specified dose.

2 Is calcium present in supplement, if yes how much.

3 Type of validation: (a) Validation DEQAS CV ≤10%; (b) Other type of validation, CV ≤12%; (c) no info on type of validation, CV ≤12%; (d) no info validation or CV%.

4 The study is judged to be at low (L) or high risk of bias (H), when at least one domain was judged to be L or H [17]. In case 2 domains were unclear instead of low, unclear (UC) is the judgment. For further information, see Supplement 3.

5 D2-D3 comparisons were based on PP data, which were comparable to ITT data as none of the randomized subjects were lost to follow-up.

6 ITT data of Glendenning et al. [28] was judged to be the data from 74% of the randomized subjects, who completed the study with a compliance of 59% in the D2-group and 47% in the D3-group (*P* = 0.33). The PP data of Glendenning et al. [28] was judged to be the data from 39% of the randomized subjects with a compliance of >80%.

### Results main analyses

**FIGURE 2:**
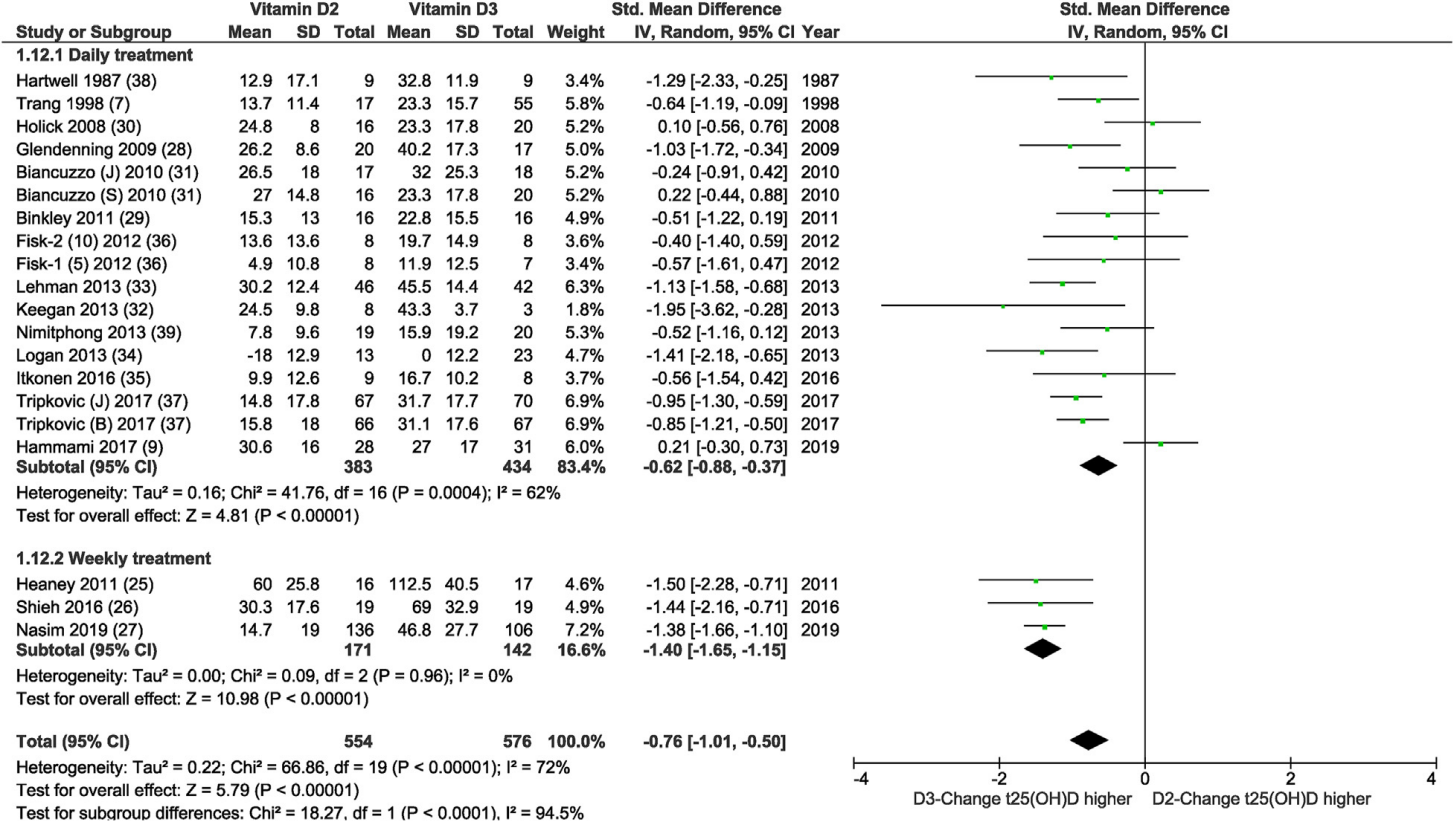
Random-effects meta-analysis comparing the effects of daily and weekly supplementation of D2 with that of D3 on net changes in serum 25(OH)D concentrations. The forest plot indicates that the absolute change in 25(OH)D from baseline favored the D3 intervention. In the figure, “vitamin D2” and “vitamin D3” denotes the change in serum 25(OH)D concentrations from baseline (net change) in the D2 and D3 group respectively, and “Total” denotes the cumulative n from all included comparisons. Using a random-effects model, there was generally a significantly smaller effect in the raising of serum 25 (OH)D concentrations over time for D2 supplementation than for D3 supplementation, which was more striking when vitamin D was administered less often (P < 0.00001). Excluding the low-quality studies [25,27,[32], [33], [34],^36^,^39^], the SMD of the subgroup consisting of studies with a daily dosing schedule was −0.49 (95% CI: −0.80, −0.18; *I*^*2*^ = 67%; *P* = 0.002). IV, inverse variance; t25(OH)D, total 25(OH)D concentration.

**FIGURE 3:**
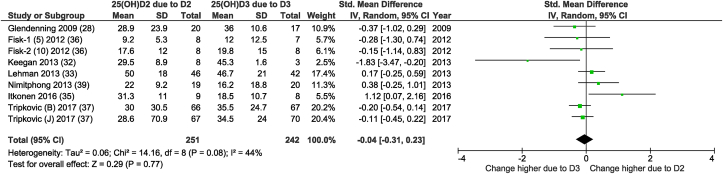
Random-effects meta-analysis comparing the effects of daily supplementation of D2 with that of D3 on the D2-induced change in 25(OH)D2 with the D3-induced change in 25(OH)D3 concentrations. The forest plot indicates that no difference in the absolute change in 25(OH)D2/3 was observed. In the figure, “25(OH)D2 due to D2” and “25(OH)D3 due to D3” denotes the vitamin D2-induced change in 25(OH)D2 and the vitamin D3-induced change in 25(OH)D3 concentrations from baseline (net change), and “Total” denotes the cumulative n from all included comparisons. As shown in Supplementary Figure S4D4, excluding the low-quality studies [32,33,36,39], the SMD was −0.07 (95% CI: −0.43, 0.28; I^2^ = 51%; *P* = 0.69). IV, inverse variance.

As shown in Figure 2, the SMD of the meta-analysis was −0.76 (95% CI: −1.01, −0.50; *I*^*2*^ = 72%; *P* < 0.00001) indicating a smaller change in total 25(OH)D in the vitamin D2 group compared with the vitamin D3 group. When comparing the vitamin D2-induced increase in 25(OH)D2 with the vitamin D3-induced increase in 25(OH)D3 involving 9 comparisons, all based on a daily dosing regimen using similar doses, no significant difference was found, and heterogeneity was moderate (Figure 3).

### Sensitivity analyses

The meta-analysis is based on data, either from PP or ITT analyses, that have been described in the main paper and which are summarized in Table 1, TABLE 2, and henceforth referred to as “mixed.” The meta-analysis included a total of 554 subjects who received vitamin D2 and 576 subjects who received vitamin D3. These numbers were 232 compared with 247 in the “ITT” meta-analysis, and 421 compared with 439 subjects in the “PP” meta-analysis. As mentioned, Figure 2 and Supplementary Figure S4A1 show the results of the “mixed” meta-analysis. The SMD of the meta-analysis using data from studies with an ITT (Supplementary Information S4C1: 7 comparisons, only daily dosing regimen) or PP meta-analyses (Supplementary Information S4A2: 18 comparisons) was −0.76 (95% CI: −1.07, −0.44; *I*^*2*^ = 58%; *P* < 0.00001) and −0.74 (95% CI: −1.05, −0.43; *I*^*2*^ = 74%; *P* < 0.00001), respectively. Similar small differences in SMD were found when limiting to studies that dosed vitamin D daily (Supplementary Figures S4B1–2, S4C1). This was also the case when comparing the vitamin D2-induced increase in 25(OH)D2 with the vitamin D3-induced increase in 25(OH)D3 (see Supplementary Figures S4D1–3). Because the outcomes of the meta-analysis based on ITT or PP analyses were comparable with the outcome of the “mixed” meta-analysis, the remaining sensitivity and all subgroup analyses were performed on “mixed” data from either PP or ITT analyses described in the main papers of the individual studies. As summarized in Table 3 and Supplementary Figures S4A3 S4B3, and S4D4, results of other sensitivity analyses on change in total 25(OH)D were similar to the main analyses. The estimated overall WMD in change in total 25(OH)D based on 12 daily dosed vitamin D2-vitamin D3 comparisons (see Supplementary Figure S4C2), analyzed using LC-MS/MS, was 10.39 nmol/L lower for the vitamin D2 group compared with the vitamin D3 group (95% CI: −14.62, −6,16; *I*^*2*^ = 64%; *P* < 00001). Multiplying the vitamin D2- or vitamin D3-induced change in total 25(OH)D by weight, obtained from the meta-analysis shown in Supplementary Figure S4C2, the difference of 10.39 nmol/L was found to be equal to 40%. Excluding the studies classified as being of “high risk” of bias [[32], [33], [34],^36^,^39^], the MD changed to −7.27 (95% CI: −14.67, 0.14; *I*^*2*^ = 77%; *P* = 0.05).

**TABLE 3:** Meta-analysis and sensitivity analyses on serum total 25(OH)D and 25(OH)D2/3 concentrations, based on all D2-D3 comparisons available, on D2-D3 comparisons obtained from studies with low to unclear risk of bias, or studies using HPLC-MS/MS analyses^1^

	Included studies^2^	SMD/MD	95% CI	*P*-value	*I*^2^ (%)	*n* D2/D3
Both daily and weekly dosed D2-D3 comparisons in changing total 25(OH)D concentration						
All studies	[[7], [9], [25], [26], [27], [28], [29], [30], [31], [32], [33], [34], [35], [36], [37], [38], [39]]	−0.76	−1.01, −0.50	<0.00001	72	554/576
Excluding high risk of bias-studies^3^	[[7], [9], [26], [28], [29], [30], [31], [35], [37], [38]]	−0.56	−0.87, −0.25	0.0004	69	300/350
Only daily dosed D2-D3 comparison in changing total 25(OH)D concentration						
All studies	[[7], [9], [28], [29], [30], [31], [32], [33], [34], [35], [36], [37], [38], [39]]	−0.62	−0.88, −0.37	<0.00001	62	383/434
Excluding high risk of bias-studies^3^	[[7], [9], [28], [29], [30], [31], [35], [37], [38]]	−0.49	−0.80, −0.18	0.002	67	281/331
Only studies analyzed using HPLC-MS/MS	[[30], [31], [32], [33], [34], [35], [36], [37], [39]]	−10.39 nmol/L	−14.62, −6.16	<0.00001	64	293/306
D2-induced change in 25(OH)D2 vs. D3-induced change in 25(OH)D3^4^						
All studies	[[28], [32], [33], [35], [36], [37], [39]]	−0.04	−0.31, 0.23	0.77	44	251/242
Excluding high risk of bias-studies^3^	[[28], [35], [37]]	−0.07	−0.43, 0.28	0.69	51	162/162

1 See Supplementary Figure S4 for the forest plots. Abbreviations: 25(OH)D, 25-hydroxyvitamin D; CI, confidence interval; *I*^*2*^, heterogeneity; MD, mean difference, shown only when studies are included that measured 25(OH)D concentrations using high-performance liquid chromatography-tandem mass spectrometry; *P*, *P* value of D2-D3 comparison; SMD, standardized mean difference.

2 Some studies [31,36,37] reported 2 instead of 1 D2-D3 comparison.

3 The study is judged to be at unclear (UC), low (L), or high risk of bias (H), when one domain was judged to be UC, L or H [17]. See Table with domains in Supplement 3.

4 Changes in 25(OH)D2 and 25(OH)D3 due to vitamin D2 and D3, respectively, are compared directly.

### Results subgroup analyses on total 25(OH)D concentration

Figure 2 shows a significant difference (*P* < 0.0001) between the vitamin D2-vitamin D3 comparisons between the subgroups dosing vitamin D daily compared with weekly. Although no heterogeneity was found in the subgroup of studies that dosed vitamin D once or twice a week [[25], [26], [27]], heterogeneity was still high in the subgroup of studies that dosed vitamin D daily, i.e., 62%. Unfortunately, 2 of the 3 weekly dosing studies [25,27] were of low quality (see Table 2 and Supplementary Table S3).

Table 4 and Supplementary Figure S5 show the results of the subgroup meta-analyses for total 25(OH)D concentration limited to studies that dosed vitamin D daily. Nine of the 12 vitamin D2-vitamin D3 comparisons [7,9,28,30,31,33,36] that described the percentage of subjects with a baseline 25(OH)D concentration of <50 nmol/L were conducted in subjects of whom >60% had a baseline 25(OH)D concentration of <50 nmol/L. No significant difference was found between this subgroup and the subgroup with studies conducted in subjects of whom <60% had serum 25(OH)D concentration <50nmol/L (*P* = 0.22). However, the vitamin D2-vitamin D3 comparison in the subgroup conducted primarily in subjects with a baseline 25(OH)D <50 nmol/L lost significance (SMD: −0.39; 95% CI: −0.77, −0.00; *I*^*2*^ = 68%; *P* = 0.05), but the heterogeneity remained substantial compared with the other subgroup (SMD: −0.83; 95% CI: −1.42, −0.24; *I*^*2*^ = 42%; *P* = 0.006). Excluding low-quality studies did not change the outcome (see Supplementary Figure S5).

**TABLE 4:** Systematic review of subgroup results for serum total 25(OH)D concentration, only including daily dosed studies^1^

All studies	SMD	95% CI	*P*^2^	*I^2^* (%)	*n* D2/D3 (#^3^)	*P* value diff^4^
% of subjects with a baseline 25(OH)D <50 nmol/L						
>60%	−0.39	−0.77, −0.00	0.05	68	176/218 (9/4)	
≤60%	−0.83	−1.42, −0.24	0.006	42	41/51 (3/0)	0.22
Race						
>50% Caucasian	−0.87	−1.08, −0.66	<0.00001	0	196/208 (8/1)	
≤50% Caucasian	−0.15	−0.46, 0.17	0.37	38	113/164 (6/4)	0.0002
Age						
<65 y	−0.77	−1.05, −0.49	<0.00001	56	298/343 (12/1)	
≥65 y	−0.28	−0.72, 0.16	0.21	52	85/91 (5/4)	0.07
Sex						
>70% women	−0.92	−1.13, −0.70	<0.00001	0	191/200 (7/0)	
≤70% women	−0.33	−0.70, 0.03	0.07	64	172/217 (9/5)	0.007
Latitude						
≥45°N	−0.91	−1.12, −0.71	<0.00001	0	213/211 (7/0)	
30 to <45°N	−0.31	−0.71, 0.09	0.13	47	90/132 (6/4)	
<30°N	−0.65	−1.38, 0.07	0.08	80	80/91 (4/1)	0.0008
Average daily dose						
≤25 μg	−0.59	−0.88, −0.30	<0.0001	56	259/278 (11/3)	
>25 μg	−0.73	−1.28, −0.18	0.009	74	124/156 (2/0)	0.66
Calcium included in D treatment						
Yes	−0.84	−1.27, −0.42	0.0001	0	48/46 (3/0)	
No	−0.57	−0.86, −0.28	0.0001	67	335/388 (14/5)	0.30

1 See Supplementary Figure 5 for the forest plots. Abbreviations: 25(OH)D, 25-hydroxyvitamin D; CI, confidence interval; *I*^*2*^, heterogeneity; RCT, randomized controlled trial; SMD, standardized mean difference.

2 *P* value of D2-D3 comparison within subgroup.

3 Number within parentheses is described as the number of D2-D3 comparisons included in the specified subgroup; behind “/” is mentioned the number of D2-D3 comparisons included in the specified subgroup that was performed predominantly in subjects with a BMI >25. For example, 4 out of 5 comparisons in the subgroup of studies conducted in subjects aged 65+ were predominantly overweight or obese.

4 *P* value of difference between subgroups.

**FIGURE 4:**
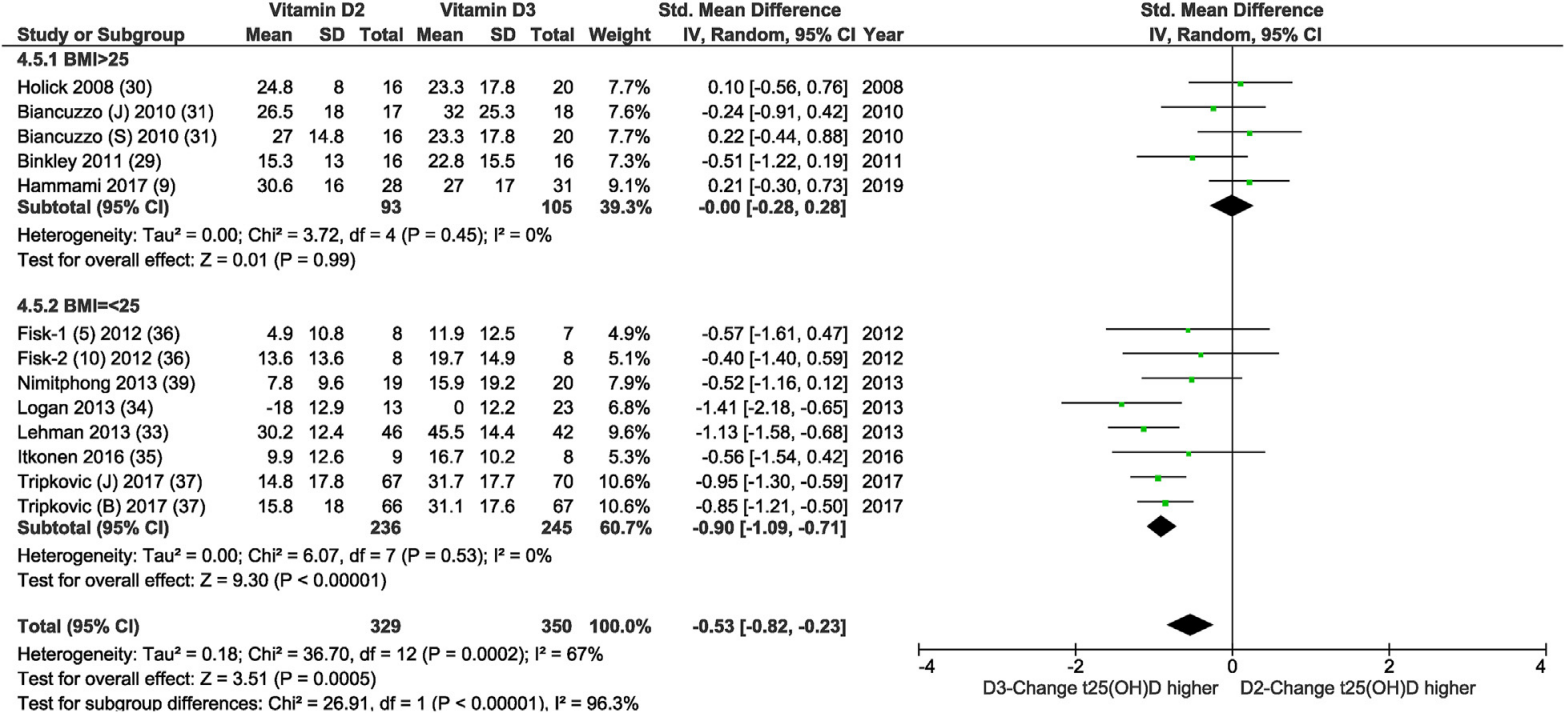
Random-effects meta-analysis comparing the effects of average BMI > 25 vs. BMI < 25 on net changes in serum 25(OH)D concentrations. In the figure, “vitamin D2” and “vitamin D3” denotes the change in serum 25(OH)D concentrations from baseline (net change) in the daily dosed D2 and D3 group respectively, and “Total” denotes the cumulative number of all included comparisons. Using a random-effects model, no significant difference was found between the raising of serum 25 (OH)D concentrations over time for D2 supplementation and for D3 supplementation in subjects with overweight or obesity, whereas in subjects with a healthy weight a significantly smaller effect was found in the raising of serum 25 (OH)D concentrations over time for D2 supplementation than for D3 supplementation. The test for subgroup differences suggests that there is a statistically significant subgroup effect (*P* < 0.00001), meaning that BMI significantly modifies the effect of the intervention. Excluding the low-quality studies [33,34,36,39] the SMD in the D2-D3 comparison in predominantly subjects with healthy weight was −0.88 (95% CI: −1.12, −0.64; I^2^ = 0%; *P* < 0.00001) with no impact on the other subgroup or the *P* value of the difference. IV, inverse variance; t25(OH)D, total 25(OH)D concentration.

As shown in Table 4 and Supplementary Figure S5, heterogeneity was lower in most subgroup analyses. When considering the subgroups based on race, age, sex, latitude, and BMI, all showed a significant difference between subgroups in the effect of vitamin D2 and vitamin D3 on total 25(OH)D concentration. However, BMI showed the strongest effect on heterogeneity toward 0% in both subgroups (see Figure 4). The SMD in the vitamin D2-vitamin D3 comparison in subjects predominantly with overweight or obesity was 0 (95% CI: −0.28, 0.28; *I*^*2*^ = 0%; *P* = 0.99) compared with −0.9 (95% CI −1.09, −0.71; *I*^*2*^ = 0%; *P* < 0.00001) in the subjects predominantly with a BMI of <25 (Figure 4). By including only studies analyzed using LC-MS/MS [[30], [31], [32], [33], [34], [35], [36], [37],^39^], the MD instead of SMD could be calculated. This resulted in an MD of the vitamin D2-vitamin D3 comparison in subjects predominantly with overweight of 0.98 nmol/L (95% CI: −5.14, 7.10 nmol/L; *I*^*2*^ = 0%; *P* = 0.75) compared with −13.77 nmol/L (95% CI: −16.75, −10.79 nmol/L; *I*^*2*^ = 11%; *P* < 0.00001) in subjects predominantly with healthy weight, respectively (*P* < 0.0001).

## Discussion

### Main results

Overall, the results based on 20 comparative studies showed vitamin D3 to be superior to vitamin D2 in raising total 25(OH)D concentrations, but vitamin D2 and vitamin D3 had a similar positive impact on their corresponding 25(OH)D hydroxylated forms. The estimated overall weighted MD in change in total 25(OH)D based on 12 daily dosed vitamin D2-vitamin D3 comparisons, analyzed using LC-MS/MS, was 10.39 nmol/L lower for the vitamin D2 group compared with the vitamin D3 group (95% CI: −14.62, −6,16; *I*^*2*^ = 64%; *P* < 00001). Limiting to studies with a daily dosing regimen, the difference in efficacy between vitamin D2 and vitamin D3 to increase total 25(OH)D became nonsignificant in the subgroup consisting of studies conducted primarily in subjects with a baseline 25(OH)D <50 nmol/L. BMI was found to be the strongest of all response modifiers examined, reducing heterogeneity to 0% in both subgroups. The vitamin D2- and vitamin D3-induced change in total 25(OH)D was significantly different in subjects with a BMI of ≤25 kg/m^2^ (*P* < 0.00001) but lost significance in the subjects predominantly with a BMI >25kg/m^2^ (*P* = 0.99). However, information on BMI was only available in 13/17 daily dosed comparisons.

### Effects on 25(OH)D hydroxylated forms

This meta-analysis also showed that daily dosed vitamin D2 and vitamin D3 had a similar positive impact on their corresp-onding 25(OH)D hydroxylated forms (Figure 3). This is in agreement with the results of Lehman et al. [33] who found that hydroxylation of vitamin D2 was comparable to hydroxylation of vitamin D3 because the increase in the specific hydroxylated forms [25(OH)D2 and 25(OH)D3] was similar in the 2 groups [33]. By comparing the vitamin D2-induced change in 25(OH)D2 concentration from baseline with the vitamin D3-induced change in 25(OH)D3, the results are less dependent on the total 25(OH)D concentration at baseline. In addition, possible methodology concerns regarding the measurement of total 25(OH)D are excluded. LC-MS/MS may not measure the 3-epimer of 25(OH)D2, and the 3-epimer of 25(OH)D3 is not chromatographically resolved from 25(OH)D3 by most routine LC–tandem MS methods. Although expected to be extremely low, the 3-epi-25(OH)D2 may be influenced by vitamin D2 supplementation as the diet also contributed to the concentration of 3-epi-25(OH)D3 in serum [40]. The absence of the 3-epimer of 25(OH)D2 in the total 25(OH)D measurement could result in a lower measurement of the vitamin D2-induced change in total 25(OH)D. Although the current meta-analysis did not confirm a difference in the vitamin D2-vitamin D3 comparison in increasing total 25(OH)D between LC-MS/MS and other methods (*P* = 0.33, data not shown), this does exclude an underestimation of the efficacy of vitamin D2.

### Subgroup analysis taking into account dosing regimen

Although only studies with a frequent dosing schedule were included in this meta-analysis, daily dosing resulted in a smaller difference between vitamin D2 and vitamin D3 in increasing 25(OH)D concentration than weekly dosing. This difference was significantly different, but there were only 3 weekly dosing studies, of which 2 had high risk of bias. In the current meta-analyses, a total of 17 unique vitamin D2-vitamin D3 comparisons were included in the subgroup on daily dosing. As compared with Balachandar et al. [5], the daily dosing subgroup included 2 more studies [9,32], and 1 was excluded [41] because the same data were already included through another study [28]. Moreover, in the subgroup analysis of Balachandar et al. [5], weekly dosing was combined with monthly dosing [29,42] and included daily dosing after a single bolus dose of vitamin D [43]. This explains the different outcomes of the current and the other meta-analysis.

The reason for the significant difference between the subgroups with daily or weekly dosing studies in the current meta-analysis might be a difference in half-life, which is shorter for 25(OH)D2 than for 25(OH)D3 [44]. However, Jones et al. [44] found that this difference was mainly present in Gambian people (*P* = 0.0007). In the United Kingdom, the half-life was not different (*P* = 0.3) [44]). The 3 weekly dosing studies were performed in 100% [25], 58% [26], or 0% [27] Caucasian subjects. Only the study of Shieh et al. [26] included black Africans (1%), but also a few daily dosing studies did include 9% to 56% black African people [7,30,31,36]. As the difference in half-life of 25(OH)D2 and 25(OH)D3 is not studied in other races, no conclusion can be made on the role of half-life in the explanation of the difference between the daily and weekly dosing. Compliance cannot explain the difference between daily and weekly because compliance was high and only slightly different between treatment groups. Higher daily doses of vitamin D were used in the weekly than daily dosing regimens (see Table 2), and the meta-analysis of Balachandar et al. [5] suggested smaller differences in the efficacy of vitamin D2 and vitamin D3 at lower doses. The molecular weight of vitamin D3 is 384 whereas for vitamin D2 it is 396, resulting in a 3% lower intake of vitamin D2. The difference in half-life of 25(OH)D2 and 25(OH)D3, molecular weight, but also the low-quality of the weekly dosing studies (see Table 2 and Supplementary Table S3) may explain the greater difference in efficacy of vitamin D2 and vitamin D3 in the weekly dosing studies.

### Subgroup analysis taking into account baseline 25(OH)D concentration

The efficacy of daily dosed vitamin D2 and vitamin D3 was not significantly different in the subgroup comprised of >60% of subjects who had a baseline 25(OH)D concentration of <50 nmol/L (*P* = 0.05). Often, total 25(OH)D consists of more 25(OH)D3, due to the contribution of vitamin D3 synthesized in skin that is absent for vitamin D2 [12]. Therefore, if the baseline concentration of serum 25(OH)D is high, the 25(OH)D3:25(OH)D2 ratio is high. This results in vitamin D2 supplementation both increasing 25(OH)D2 and decreasing 25(OH)D3, which was also found by others [11,28] and in the meta-analyses of Cashman et al. [10] on UV-exposed mushrooms. The higher the baseline, the greater the vitamin D2-induced reduction of 25(OH)D3, which leads to a lower increase in total 25(OH)D and therefore a larger difference in the efficacy of vitamin D2 and vitamin D3. This might be due to induction of 24-hydroxylase leading to catabolism of 25(OH)D3, a preferential 25-hydroxylation of vitamin D2 upon increased intake of this vitamer, or that the increased vitamin D2 intake may simply dilute vitamin D3 at serum 25(OH)D and 1,25-dihydroxyvitamin D concentrations [10]. When total 25(OH)D at baseline is low, less 25(OH)3 is present, and the balancing of total 25(OH)D by a vitamin D2-induced decrease in 25(OH)D3 concentration occurs less often. Consequently, this may lead to a smaller difference in the efficacy of vitamin D2 and vitamin D3, which may explain our results.

### Subgroup analysis taking into account BMI

BMI was a significant modifier in the daily dosed vitamin D2-vitamin D3 comparisons; subjects with overweight or obesity showed no differences between vitamin D2 and vitamin D3 in raising 25(OH)D. In addition, BMI reduced heterogeneity to zero in both subgroups. However, information on BMI was only available in 13/17 daily dosed comparisons. Other subgroups, based on race, age, sex, and latitude of vitamin D, also showed a significant difference in the vitamin D2-vitamin D3 comparison in raising 25(OH)D. The subgroups with the lowest nonsignificant SMD, consisting of fewer Caucasian, more older or female subjects, or subjects living at latitude of <45°N appeared to consist mainly of subjects with a high BMI. This indicates that BMI seems a stronger modifier than race, age, sex, or latitude of vitamin D. An explanation might be that a higher BMI can lead to lower baseline 25(OH)D concentrations [21,45], which is itself is associated with a greater response to vitamin D supplement [6,19]. As described earlier, high baseline vitamin D status may differently affect vitamin D2 and vitamin D3 efficacy, which might be absent in subjects with a high BMI. In addition, the modifying nature of BMI may be explained by the relatively lower affinity of D binding protein to vitamin D2 and 25(OH)D2 [1], which makes them more accessible to extravascular tissues. In contrast to our meta-analysis, Hammami et al. [9] studied both D vitamins and found that BMI was a significant inverse response predictor to vitamin D2 but not vitamin D3. However, this was the case only during the first 4 wk of 20-wk treatment, and in the current analyses, the studies in the subgroup with subjects predominantly with overweight or obesity all lasted 11 wk or longer. Previously, for both vitamin D2 and vitamin D3, a negative association was found between the 25(OH)D response and BMI [6,46]; the response depended on both BMI and baseline vitamin D concentration [6]. Whether there is a difference in body fat distribution between vitamin D2 and vitamin D3 needs further study. However, a lower baseline 25(OH)D and thus a lower 25(OH)D3:25(OH)D2 ratio and vitamin D2-induced reduction of 25(OH)D3 concentration could, at least partly, explain the differences in subgroups based on BMI, as all studies conducted predominantly in subjects with overweight or obesity also consisted predominantly of subjects who had baseline 25(OH)D concentration of <50 nmol/L (see Table 4).

### Strengths and limitations of meta-analysis

Besides the systematic reviewing process, the strength of the current study is its focus on daily dosing studies excluding bolus dosing. A large number of unique vitamin D2-vitamin D3 comparisons are included that allowed analyses of heterogeneity and therefore provided important insights in the targeting and application of vitamin D. Compliance was good in all studies. The main limitation is lack of access to individual data, and therefore, an individual data analysis was not possible. A subgroup analysis with many subgroups might lead to false-positive results, therefore all subgroup analyses were already prespecified in PROSPERO (CRD42021272674) before the start of the analyses. The subgroup analyses might be affected by publication bias because most subgroups contain <10 vitamin D2-vitamin D3 comparisons. Some data are missing, e.g., the percentage of participants with baseline <50 nmol/L 25(OH)D was not described or provided on request for 5 of 17 vitamin D2-vitamin D3 comparisons. As shown in Figure 4, 13 of the 17 vitamin D2-vitamin D3 comparisons reported BMI. Assuming that the 4 studies not describing BMI [7,28,32,38] mainly included subjects with a healthy weight, the outcome remained the same (*P* value of difference < 0.00001), although heterogeneity increased from 0% to 30% in the subgroup with studies predominantly composed of subjects with a healthy weight. When omitting the study with an average BMI of 25.3 [9], i.e., just above 25, the outcome also remained the same (*P* value of difference < 0.0001). This suggests the modifying character of BMI is quite robust. Other missing potentially modifying factors affecting vitamin D metabolism are the intake of protein, B vitamins [12], and magnesium [47]. Magnesium affects the metabolism of vitamin D2 and vitamin D3 differently at higher vitamin D status [48] and therefore is worth studying when comparing vitamin D2 with vitamin D3.

## Conclusion

Vitamin D3 leads to a greater increase of serum 25(OH)D than vitamin D2, even if limited to daily dose studies, but vitamin D2 and vitamin D3 had similar positive impacts on their corresponding 25(OH)D hydroxylated forms. BMI and baseline 25(OH)D concentration should be considered when comparing the effect of daily vitamin D2 and vitamin D3 supplementation on total serum 25-hydroxyvitamin D concentration. Further investigation is needed to determine whether the possible interference of BMI in the comparison of D2 and D3 is (partially) independent from baseline 25(OH)D.

## Acknowledgments

T Sanders and C Fisk, M Hammami, S Itkonen, R Vieth, and U Spielauand J Dierkes kindly provided us details on the following studies in order of listing: Fisk et al. [36], Hammami et al. [9], Itkonen et al. [35], Trang et al. [7], and Lehman et al. [33]. Last but not least, we thank Dr Andrea Darling for her advice regarding publication bias. SLN gratefully acknowledges the support of the UK Biotechnology and Biological Sciences Research Council (BBSRC); with funding by the BBSRC Institute Strategic Programme Food Microbiome and Health BB/X011054/1 and its partner project BB/X020029/1.

### Author contributions

The authors’ responsibilities were as follows—EvdH, PL, NMvS: designed research (project conception, development of overall research plan, and study oversight); LJS: searched for papers; EvdH, NMvS: independently screened all studies for eligibility; EvdH: extracted all data; SALN, PL: extracted and verified all data; EvdH: performed statistical analyses and wrote paper; EvdH, NMvS, PL, SALN: had primary responsibility for final content; and all authors: read and approved the final version of the manuscript.

### Conflict of interest

EvdH is employed by Scelta Mushrooms B.V., which sells vitamin D2-enriched mushroom products. No mushrooms are included in the present meta-analysis because these products also contain other components that might interfere with the outcome. PL received a travel fee from Abiogen. SALN is a member of the UK’s H.M. Government, the Scientific Advisory Committee on Nutrition (SACN) Committee and was a member of the SACN Vitamin D Working Group (2010–2016). SALN is also on the European Food Safety Authority (EFSA) Committee on the Tolerable Upper Limit for Vitamin D. She is Research Director of D3Tex Ltd, which holds the UK and Gulf Corporation Council Patents for the use of UVB material for combating vitamin D deficiency in women who dress for cultural style. SALN has received funding from the UK Government for vitamin D2 and vitamin D3 and the European Union for vitamin D randomized controlled trials. All other authors report no conflicts of interest.

### Funding

The authors reported no funding received for this study.

### Data availability

Data not shown in the manuscript or supplementary material (e.g., funnel plots) are available from the first author.

## References

[1] Houghton L.A., Vieth R. (2006). The case against ergocalciferol (vitamin D2) as a vitamin supplement. Am. J. Clin. Nutr..

[2] Ross A.C., Taylor C.L., Yaktine A.L., del Valle H.B. (2011). https://www.ncbi.nlm.nih.gov/books/NBK56061/.

[3] Cashman K.D., Kinsella M., McNulty B.A., Walton J., Gibney M.J., Flynn A. (2014). Dietary vitamin D2--a potentially underestimated contributor to vitamin D nutritional status of adults?. Br. J. Nutr..

[4] Tripkovic L., Lambert H., Hart K., Smith C.P., Bucca G., Penson S. (2012). Comparison of vitamin D2 and vitamin D3 supplementation in raising serum 25-hydroxyvitamin D status: a systematic review and meta-analysis. Am. J. Clin. Nutr..

[5] Balachandar R., Pullakhandam R., Kulkarni B., Sachdev H.S. (2021). Relative efficacy of vitamin D2 and vitamin D3 in improving vitamin D status: systematic review and meta-analysis. Nutrients.

[6] Zwart S.R., Mehta S.K., Ploutz-Snyder R., Bourbeau Y.V., Locke J.P., Pierson D.L. (2011). Response to vitamin D supplementation during Antarctic winter is related to BMI, and supplementation can mitigate Epstein-Barr virus reactivation. J. Nutr..

[7] Trang H.M., Cole D.E.C., Rubin L.A., Pierratos A., Siu S., Vieth R. (1998). Evidence that vitamin D3 increases serum 25-hydroxyvitamin D more efficiently than does vitamin D2. Am. J. Clin. Nutr..

[8] Zajac I.T., Barnes M., Cavuoto P., Wittert G., Noakes M. (2020). The effects of vitamin D-enriched mushrooms and vitamin D3 on cognitive performance and mood in healthy elderly adults: a randomised, double-blinded, placebo-controlled trial. Nutrients.

[9] Hammami M.M., Yusuf A. (2017). Differential effects of vitamin D2 and D3 supplements on 25-hydroxyvitamin D level are dose, sex, and time dependent: a randomized controlled trial. BMC Endocr. Disord..

[10] Cashman K.D., Kiely M., Seamans K.M., Urbain P. (2016). Effect of ultraviolet light-exposed mushrooms on vitamin D status: liquid chromatography-tandem mass spectrometry reanalysis of biobanked sera from a randomized controlled trial and a systematic review plus meta-analysis. J. Nutr..

[11] Hammami M.M., Abuhdeeb K., Hammami S., Yusuf A. (2019). Vitamin-D2 treatment-associated decrease in 25(OH)D3 level is a reciprocal phenomenon: a randomized controlled trial. BMC Endocr. Disord..

[12] Cashman K.D., van den Heuvel E.G.H.M., Schoemaker R.J.W., Prévéraud D.P., Macdonald H.M., Arcot J. (2017). 25-hydroxyvitamin D as a biomarker of vitamin D status and its modeling to inform strategies for prevention of vitamin D deficiency within the population. Adv. Nutr..

[13] Page M.J., McKenzie J.E., Bossuyt P.M., Boutron I., Hoffmann T.C., Mulrow C.D. (2021). The PRISMA 2020 statement: an updated guideline for reporting systematic reviews. BMJ.

[14] Otten R., de Vries R., Schoonmade L. (2019). Amsterdam Efficient Deduplication (AED) method (version 1). https://zenodo.org/record/3582928.

[15] Bramer W.M., Giustini D., de Jonge G.B., Holland L., Bekhuis T. (2016). De-duplication of database search results for systematic reviews in EndNote. J. Med. Libr. Assoc..

[16] Ouzzani M., Hammady H., Fedorowicz Z., Elmagarmid A. (2016). Rayyan-a web and mobile app for systematic reviews. Syst. Rev..

[17] Higgins J.P.T.J., Thomas J., Chandler J., Cumpston M., Li T., Page M. (2022). https://training.cochrane.org/handbook/.

[18] Higgins J.P.T., Altman D.G., Gøtzsche P.C., Jüni P., Moher D., Oxman A.D. (2011). The Cochrane Collaboration’s tool for assessing risk of bias in randomised trials. BMJ.

[19] Best C.M., Zelnick L.R., Thummel K.E., Hsu S., Limonte C., Thadhani R. (2022). Serum vitamin D: correlates of baseline concentration and response to supplementation in VITAL-DKD. J. Clin. Endocrinol. Metab..

[20] Lips P., Duong T., Oleksik A., Black D., Cummings S., Cox D., Nickelsen T. (2001). A global study of vitamin D status and parathyroid function in postmenopausal women with osteoporosis: baseline data from the multiple outcomes of raloxifene evaluation clinical trial. J. Clin. Endocrinol. Metab..

[21] Snijder M.B., van Dam R.M., Visser M., Deeg D.J.H., Dekker J.M., Bouter L.M. (2005). Adiposity in relation to vitamin D status and parathyroid hormone levels: a population-based study in older men and women. J. Clin. Endocrinol. Metab..

[22] Dewansingh P., Reckman G.A.R., Mijlius C.F., Krijnen W.P., van der Schans C.P., Jager-Wittenaar H. (2021). Protein, calcium, vitamin D intake and 25(OH)D status in normal weight, overweight, and obese older adults: a systematic review and meta-analysis. Front. Nutr..

[23] Jorde R., Grimnes G. (2020). Increased calcium intake is associated lower serum 25-hydroxyvitamin D levels in subjects with adequate vitamin D intake: a population-based observational study. BMC Nutr.

[24] Richardson M., Garner P., Donegan S. (2019). Interpretation of subgroup analyses in systematic reviews: a tutorial. Clin. Epidemiol. Glob. Health.

[25] Heaney R.P., Recker R.R., Grote J., Horst R.L., Armas L.A.G. (2011). Vitamin D_3_ is more potent than vitamin D_2_ in humans. J. Clin. Endocrinol. Metab..

[26] Shieh A., Chun R.F., Ma C., Witzel S., Meyer B., Rafison B. (2016). Effects of high-dose vitamin D2 versus D3 on total and free 25-hydroxyvitamin D and markers of calcium balance. J. Clin. Endocrinol. Metab..

[27] Nasim B., Al Sughaiyer H., Abdul Rahman S., Inamdar B.R., Chakaki R., Abuhatab S. (2019). Efficacy of vitamin D3 versus vitamin D2 in deficient and insufficient patients: an open-label, randomized controlled trial. Ibnosina J. Med. Biomed. Sci..

[28] Glendenning P., Chew G.T., Seymour H.M., Gillett M.J., Goldswain P.R., Inderjeeth C.A. (2009). Serum 25-hydroxyvitamin D levels in vitamin D-insufficient hip fracture patients after supplementation with ergocalciferol and cholecalciferol. Bone.

[29] Binkley N., Gemar D., Engelke J., Gangnon R., Ramamurthy R., Krueger D. (2011). Evaluation of ergocalciferol or cholecalciferol dosing, 1,600 IU daily or 50,000 IU monthly in older adults. J. Clin. Endocrinol. Metab..

[30] Holick M.F., Biancuzzo R.M., Chen T.C., Klein E.K., Young A., Bibuld D. (2008). Vitamin D2 is as effective as vitamin D3 in maintaining circulating concentrations of 25-hydroxyvitamin D. J. Clin. Endocrinol. Metab..

[31] Biancuzzo R.M., Young A., Bibuld D., Cai M.H., Winter M.R., Klein E.K. (2010). Fortification of orange juice with vitamin D_2_ or vitamin D_3_ is as effective as an oral supplement in maintaining vitamin D status in adults. Am. J. Clin. Nutr..

[32] Keegan R.J.H., Lu Z., Bogusz J.M., Williams J.E., Holick M.F. (2013). Photobiology of vitamin D in mushrooms and its bioavailability in humans. Dermatoendocrinol.

[33] Lehmann U., Hirche F., Stangl G.I., Hinz K., Westphal S., Dierkes J. (2013). Bioavailability of vitamin D_2_ and D_3_ in healthy volunteers, a randomized placebo-controlled trial. J. Clin. Endocrinol. Metab..

[34] Logan V.F., Gray A.R., Peddie M.C., Harper M.J., Houghton L.A. (2013). Long-term vitamin D3 supplementation is more effective than vitamin D2 in maintaining serum 25-hydroxyvitamin D status over the winter months. Br. J. Nutr..

[35] Itkonen S.T., Skaffari E., Saaristo P., Saarnio E.M., Erkkola M., Jakobsen J. (2016). Effects of vitamin D2-fortified bread v. supplementation with vitamin D2 or D3 on serum 25-hydroxyvitamin D metabolites: an 8-week randomised-controlled trial in young adult Finnish women. Br. J. Nutr..

[36] Fisk C.M., Theobald H.E., Sanders T.A.B. (2012). Fortified malted milk drinks containing low-dose ergocalciferol and cholecalciferol do not differ in their capacity to raise serum 25-hydroxyvitamin D concentrations in healthy men and women not exposed to UV-B. J. Nutr..

[37] Tripkovic L., Wilson L.R., Hart K., Johnsen S., de Lusignan S., Smith C.P. (2017). Daily supplementation with 15 μg vitamin D2 compared with vitamin D3 to increase wintertime 25-hydroxyvitamin D status in healthy South Asian and white European women: a 12-wk randomized, placebo-controlled food-fortification trial. Am. J. Clin. Nutr..

[38] Hartwell D., Hassager C., Christiansen C. (1987). Effect of vitamin D2 and vitamin D3 on the serum concentrations of 1,25(OH)2D2, and 1,25(OH)2D3 in normal subjects. Acta Endocrinol.

[39] Nimitphong H., Saetung S., Chanprasertyotin S., Chailurkit L.O., Ongphiphadhanakul B. (2013). Changes in circulating 25-hydroxyvitamin D according to vitamin D binding protein genotypes after vitamin D₃ or D₂ supplementation. Nutr. J..

[40] Cashman K.D., Kinsella M., Walton J., Flynn A., Hayes A., Lucey A.J. (2014). The 3 epimer of 25-hydroxycholecalciferol is present in the circulation of the majority of adults in a nationally representative sample and has endogenous origins. J. Nutr..

[41] Glendenning P., Chew G.T., Inderjeeth C.A., Taranto M., Fraser W.D. (2013). Calculated free and bioavailable vitamin D metabolite concentrations in vitamin D-deficient hip fracture patients after supplementation with cholecalciferol and ergocalciferol. Bone.

[42] Martineau A.R., Thummel K.E., Wang Z., Jolliffe D.A., Boucher B.J., Griffin S.J. (2019). Differential effects of oral boluses of vitamin D2 vs vitamin D3 on vitamin D metabolism: a randomized controlled trial. J. Clin. Endocrinol. Metab..

[43] Oliveri B., Mastaglia S.R., Brito G.M., Seijo M., Keller G.A., Somoza J. (2015). Vitamin D3 seems more appropriate than D2 to sustain adequate levels of 25OHD: a pharmacokinetic approach. Eur. J. Clin. Nutr..

[44] Jones K.S., Assar S., Harnpanich D., Bouillon R., Lambrechts D., Prentice A. (2014). 25(OH)D2 half-life is shorter than 25(OH)D3 half-life and is influenced by DBP concentration and genotype. J. Clin. Endocrinol. Metab..

[45] Mallard S.R., Howe A.S., Houghton L.A. (2016). Vitamin D status and weight loss: a systematic review and meta-analysis of randomized and nonrandomized controlled weight-loss trials. Am. J. Clin. Nutr..

[46] Binkley N., Borchardt G., Siglinsky E., Krueger D. (2017). Does vitamin D metabolite measurement help predict 25(OH)D change following vitamin D supplementation?. Endocr. Pract..

[47] Deng X., Song Y., Manson J.E., Signorello L.B., Zhang S.M., Shrubsole M.J. (2013). Magnesium, vitamin D status and mortality: results from US National Health and Nutrition Examination Survey (NHANES) 2001 to 2006 and NHANES III. BMC Med.

[48] Dai Q., Zhu X., Manson J.A.E., Song Y., Li X., Franke A.A. (2018). Magnesium status and supplementation influence vitamin D status and metabolism: results from a randomized trial. Am. J. Clin. Nutr..

