# Supplementary material for: Comparison of the Effect of Daily Vitamin D2 and Vitamin D3 Supplementation on Serum 25-Hydroxyvitamin D Concentration (Total 25(OH)D, 25(OH)D2, and 25(OH)D3) and Importance of Body Mass Index: A Systematic Review and Meta-Analysis
Source: Adv Nutr. 2023 Oct 20;15(1):100133. doi: 10.1016/j.advnut.2023.09.016 (PMC10831883; doi:10.1016/j.advnut.2023.09.016)

Comparison of the effect of daily vitamin D2 and vitamin D3 supplementation on serum 25-hydroxyvitamin D concentration (total 25(OH)D, 25(OH)D2 and 25(OHD3) and importance of body mass index: a systematic review and meta-analysis. Ellen GHM van den Heuvel et al.

Supplementary Table S1

PubMed History and Search Details June 7, 2022

Search	PubMed Query – June 7, 2022	Results
#4	**#1 AND #2 AND #3**	352
#3	"Randomized Controlled Trial" [Publication Type] OR "Controlled Clinical Trial" [Publication Type] OR "Clinical Trials as Topic"[Mesh] OR trial[tiab] OR randomized[tiab] OR randomised[tiab] OR randomly[tiab] OR rct[tiab]	1,747,887
#2	"Cholecalciferol"[Mesh] OR cholecalciferol*[tiab] OR "vitamin D3"[tiab] OR "vitamin D 3"[tiab]	34,167
#1	"Ergocalciferols"[Mesh] OR ergocalciferol*[tiab] OR "vitamin D2"[tiab] OR "vitamin D 2"[tiab]	5,581

Embase History and Search Details June 7, 2022

Search	Embase.com Query – June 7, 2022	Results
#5	#4 NOT 'conference abstract'/it	691
#4	**#1 AND #2 AND #3**	777
#3	'clinical trial'/exp OR 'randomized controlled trial'/exp OR (trial OR randomized OR randomised OR randomly OR rct):ab,ti,kw	2,812,836
#2	'colecalciferol'/exp OR (cholecalciferol* OR colecalciferol* OR ‘vitamin D3’ OR ‘vitamin D 3’):ab,ti,kw	34,766
#1	'ergocalciferol'/exp OR (ergocalciferol* OR ‘vitamin D2’ OR ‘vitamin D 2’):ab,ti,kw	11,007

Web of Science Core Collection History and Search Details June 7, 2022

Search	Web of Science Core Collection Query – June 7, 2022	Results
#4	#1 AND #2 AND #3	226
#3	TS = (“trial” OR “randomized” OR “randomised” OR “randomly” OR rct)	2,000,259
#2	TS = (cholecalciferol* OR colecalciferol* OR “vitamin D3” OR “vitamin D 3”)	18,167
#1	TS = (ergocalciferol* OR “vitamin D2” OR “vitamin D 2”)	2,349

The Cochrane Library History and Search Details June 7, 2022

Search	The Cochrane Library (Wiley) Query – June 7, 2022	Results
#5	Limit to: Trials	528
#4	#1 and #2 and #3	533
#3	(bioav* OR D-status OR "D status" OR 250HD* OR (25 NEAR/3 D*)):ti,ab,kw<br>(Word variations have been searched)	64,807
#2	(cholecalciferol* OR colecalciferol* OR “vitamin D3” OR “vitamin D 3”):ti,ab,kw<br>(Word variations have been searched)	5,769
#1	(ergocalciferol* OR “vitamin D2” OR “vitamin D 2”):ti,ab,kw<br>(Word variations have been searched)	1,904

Supplementary Figure S2 Flowchart of the search and selection procedure of studies

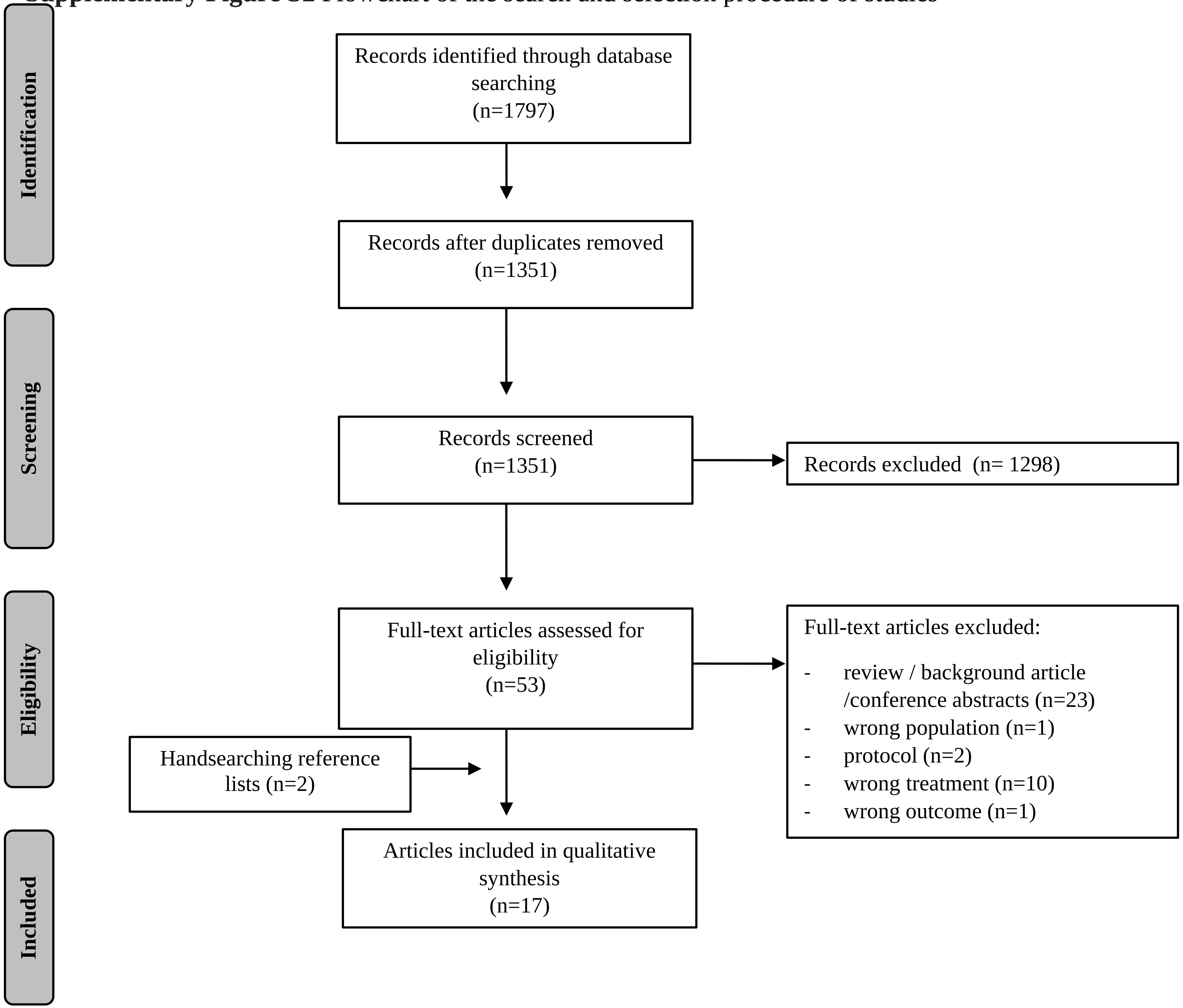

Supplementary Table S3. Risk of bias for individual studies assessed according to Cochrane Group (Higgins et al, BMJ 2011)

	Random sequence generation	Allocation concealment	Blinding of participants and personnel	Blinding of outcome assessment	Incomplete outcome data	Selective reporting	Other sources of bias
Hartwell (38)	low	unclear	unclear	unclear	low	unclear	unclear[a]
Trang (7)	low	unclear	low	unclear	low	unclear	low
Holick (30)	low	unclear	low	unclear	low	low	low
Glendenning (28)	low	low	low	unclear	low	unclear	unclear[a]
Binkley (29)	low	unclear	low	unclear	low	low	low
Heaney (25)	low	high	high	unclear	low	low	high[a9]
Lehman (33)	low	Unclear	low	low	low	low	high[a6]
Nimitphong (39)	low	high	high	unclear	low	low	unclear[a], high[c]
Logan (34)	low	unclear	low	low	low	low	high[a4]
Keegan (32)	low	unclear	unclear	unclear	high	unclear	low
Itkonen (35)	low	low	low	unclear	low	low	low
Shieh (26)	low	unclear	unclear	unclear	low	low	low
Hammami (9)	low	unclear	low	low	low	low	low
Nasim (27)	low	high	high	high	low	low	unclear[a,b]
Biancuzzo-S (31)	low	unclear	low	unclear	low	low	low
Biancuzzo-J (31)	low	unclear	low	unclear	low	low	low
Fisk-5 (36)	low	low	low	low	low	low	high[b]
Fisk-10 (36)	low	low	low	low	low	low	high[a2.5,b]
Tripkovic-J (37)	low	low	low	low	low	low	low
Tripkovic-B (37)	low	low	low	low	low	low	low

Other sources of bias are related to whether groups treated equally, apart from the intervention: [a] difference in analyzed content of D2 vs. D3 >10% of total dose, also mentioned is the difference in micrograms; [b] baseline 25(OH)D between D2 and D3 treated groups differ >20% of average; [c] Ca in supplement different quantity >200 mg/d

Supplementary Figures S4. Sensitivity analyses. “Total” denotes the cumulative number of all included comparisons; IV, inverse variance; t25(OH)D, total 25(OH)D concentration; Trials are sorted out by dosing of study and outcome: A. Daily and weekly dosing regimen; B. Only daily dosing regimen is included; C. & D. Only daily dosing regimen is available and included. In Figures A, B and C, “vitamin D2” and “vitamin D3” denotes the change in serum 25(OH)D concentrations from baseline (net change) in the D2 and D3 group respectively. In the Figures D, change in 25(OH)D2 and 25(OH)D3 due to vitamin D2 and D3, respectively, are compared directly. Figure A1, B1 and D1 include mixed data, either from per protocol or intention-to-treat analyses; Figures A2, B2 and D2 included data from per protocol analyses; Figures C1 and D3 data from intention-to-treat analyses; Figure A3, B3, D4 exclude studies classified as being of ‘high risk’ of bias; and Figure C2 include daily dosing studies using HPLC-MS/MS analyses.

A1

Study or Subgroup	Vitamin D2 Mean	Vitamin D2 SD	Vitamin D2 Total	Vitamin D3 Mean	Vitamin D3 SD	Vitamin D3 Total	Weight	Std. Mean Difference IV, Random, 95% CI	Year
Hartwell 1987 (38)	12.9	17.1	9	32.8	11.9	9	3.4%	-1.29 [-2.33, -0.25]	1987
Trang 1998 (7)	13.7	11.4	17	23.3	15.7	55	5.8%	-0.64 [-1.19, -0.09]	1998
Holick 2008 (30)	24.8	8	16	23.3	17.8	20	5.2%	0.10 [-0.56, 0.76]	2008
Glendenning 2009 (28)	26.2	8.6	20	40.2	17.3	17	5.0%	-1.03 [-1.72, -0.34]	2009
Biancuzzo (S) 2010 (31)	27	14.8	16	23.3	17.8	20	5.2%	0.22 [-0.44, 0.88]	2010
Biancuzzo (J) 2010 (31)	26.5	18	17	32	25.3	18	5.2%	-0.24 [-0.91, 0.42]	2010
Binkley 2011 (29)	15.3	13	16	22.8	15.5	16	4.9%	-0.51 [-1.22, 0.19]	2011
Heaney 2011 (25)	60	25.8	16	112.5	40.5	17	4.6%	-1.50 [-2.28, -0.71]	2011
Fisk-1 (5) 2012 (36)	4.9	10.8	8	11.9	12.5	7	3.4%	-0.57 [-1.61, 0.47]	2012
Fisk-2 (10) 2012 (36)	13.6	13.6	8	19.7	14.9	8	3.6%	-0.40 [-1.40, 0.59]	2012
Keegan 2013 (32)	24.5	9.8	8	43.3	3.7	3	1.8%	-1.95 [-3.62, -0.28]	2013
Lehman 2013 (33)	30.2	12.4	46	45.5	14.4	42	6.3%	-1.13 [-1.58, -0.68]	2013
Logan 2013 (34)	-18	12.9	13	0	12.2	23	4.7%	-1.41 [-2.18, -0.65]	2013
Nimitphong 2013 (39)	7.8	9.6	19	15.9	19.2	20	5.3%	-0.52 [-1.16, 0.12]	2013
Itkonen 2016 (35)	9.9	12.6	9	16.7	10.2	8	3.7%	-0.56 [-1.54, 0.42]	2016
Shieh 2016 (26)	30.3	17.6	19	69	32.9	19	4.9%	-1.44 [-2.16, -0.71]	2016
Tripkovic (J) 2017 (37)	14.8	17.8	67	31.7	17.7	70	6.9%	-0.95 [-1.30, -0.59]	2017
Tripkovic (B) 2017 (37)	15.8	18	66	31.1	17.6	67	6.9%	-0.85 [-1.21, -0.50]	2017
Hammami 2017 (9)	30.6	16	28	27	17	31	6.0%	0.21 [-0.30, 0.73]	2019
Nasim 2019 (27)	14.7	19	136	46.8	27.7	106	7.2%	-1.38 [-1.66, -1.10]	2019
**Total (95% CI)**			**554**			**576**	**100.0%**	**-0.76 [-1.01, -0.50]**	

Heterogeneity: Tau² = 0.22; Chi² = 66.86, df = 19 (P < 0.00001); I² = 72%
Test for overall effect: Z = 5.79 (P < 0.00001)

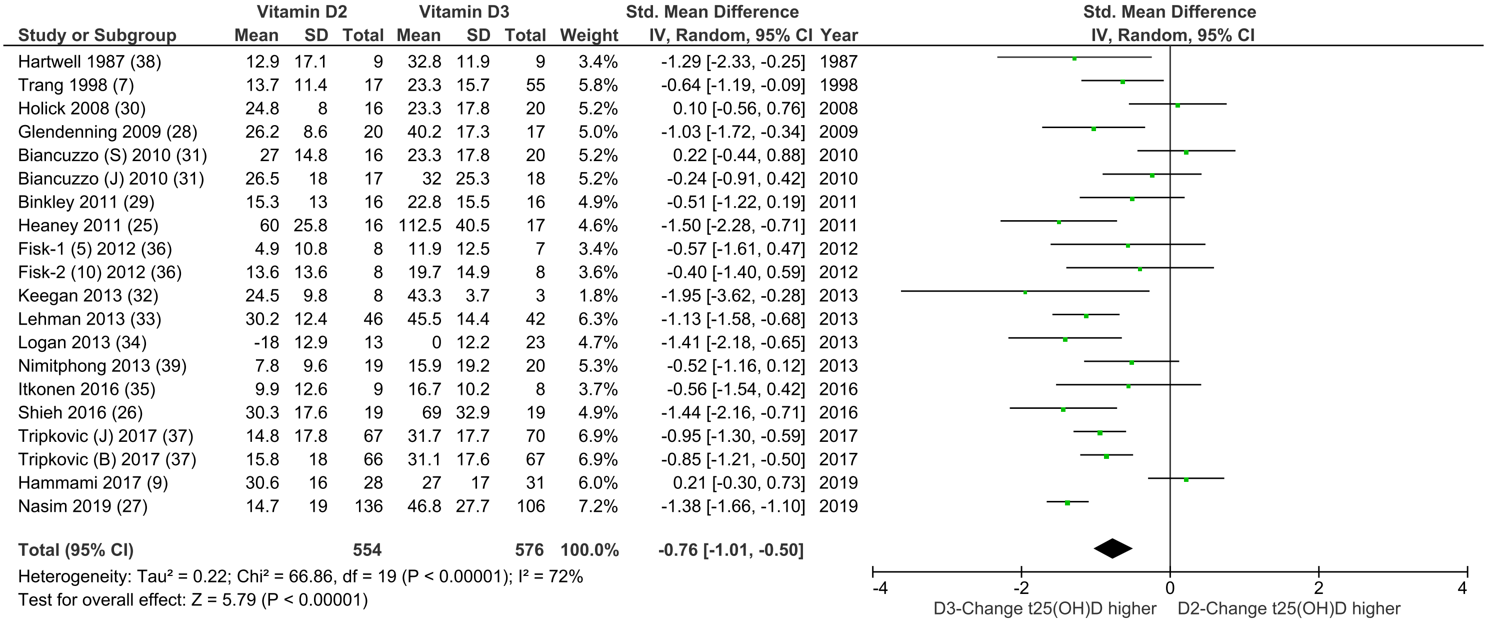

A2

Study or Subgroup	Vitamin D2 Mean	SD	Total	Vitamin D3 Mean	SD	Total	Weight	Std. Mean Difference IV, Random, 95% CI	Year
Hartwell 1987 (38)	12.9	17.1	9	32.8	11.9	9	4.3%	-1.29 [-2.33, -0.25]	1987
Trang 1998 (7)	13.7	11.4	17	23.3	15.7	55	6.5%	-0.64 [-1.19, -0.09]	1998
Holick 2008 (30)	24.8	8	16	23.3	17.8	20	6.0%	0.10 [-0.56, 0.76]	2008
Glendenning 2009 (28)	26.2	8.6	20	40.2	17.3	17	5.8%	-1.03 [-1.72, -0.34]	2009
Biancuzzo (S) 2010 (31)	27	14.8	16	23.3	17.8	20	6.0%	0.22 [-0.44, 0.88]	2010
Biancuzzo (J) 2010 (31)	26.5	18	17	32	25.3	18	6.0%	-0.24 [-0.91, 0.42]	2010
Binkley 2011 (29)	15.3	13	16	22.8	15.5	16	5.8%	-0.51 [-1.22, 0.19]	2011
Heaney 2011 (25)	60	25.8	16	112.5	40.5	17	5.4%	-1.50 [-2.28, -0.71]	2011
Fisk-1 (5) 2012 (36)	4.9	10.8	8	11.9	12.5	7	4.3%	-0.57 [-1.61, 0.47]	2012
Fisk-2 (10) 2012 (36)	13.6	13.6	8	19.7	14.9	8	4.5%	-0.40 [-1.40, 0.59]	2012
Nimitphong 2013 (39)	7.8	9.6	19	15.9	19.2	20	6.1%	-0.52 [-1.16, 0.12]	2013
Keegan 2013 (32)	24.5	9.8	8	43.3	3.7	3	2.4%	-1.95 [-3.62, -0.28]	2013
Lehman 2013 (33)	30.2	12.4	46	45.5	14.4	42	7.0%	-1.13 [-1.58, -0.68]	2013
Logan 2013 (34)	-18	12.9	13	0	12.2	23	5.5%	-1.41 [-2.18, -0.65]	2013
Itkonen 2016 (35)	9.9	12.6	9	16.7	10.2	8	4.5%	-0.56 [-1.54, 0.42]	2016
Shieh 2016 (26)	30.3	17.6	19	69	32.9	19	5.7%	-1.44 [-2.16, -0.71]	2016
Hammami 2017 (9)	30.6	16	28	27	17	31	6.7%	0.21 [-0.30, 0.73]	2019
Nasim 2019 (27)	14.7	19	136	46.8	27.7	106	7.7%	-1.38 [-1.66, -1.10]	2019
**Total (95% CI)**			**421**			**439**	**100.0%**	**-0.74 [-1.05, -0.43]**	

Heterogeneity: Tau² = 0.31; Chi² = 66.50, df = 17 (P < 0.00001); I² = 74%
Test for overall effect: Z = 4.66 (P < 0.00001)

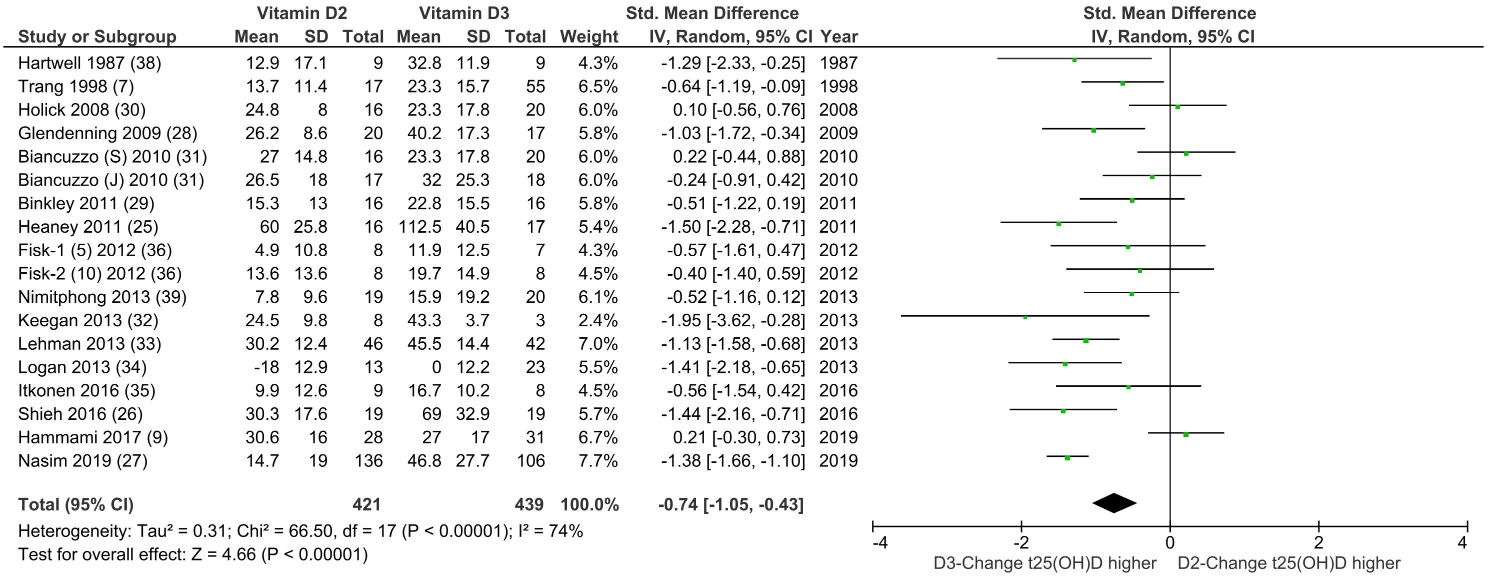

A3

Study or Subgroup	Vitamin D2 Mean	Vitamin D2 SD	Vitamin D2 Total	Vitamin D3 Mean	Vitamin D3 SD	Vitamin D3 Total	Weight	Std. Mean Difference IV, Random, 95% CI	Year
Hartwell 1987 (38)	12.9	17.1	9	32.8	11.9	9	5.3%	-1.29 [-2.33, -0.25]	1987
Trang 1998 (7)	13.7	11.4	17	23.3	15.7	55	9.2%	-0.64 [-1.19, -0.09]	1998
Holick 2008 (30)	24.8	8	16	23.3	17.8	20	8.2%	0.10 [-0.56, 0.76]	2008
Glendenning 2009 (28)	26.2	8.6	20	40.2	17.3	17	7.9%	-1.03 [-1.72, -0.34]	2009
Biancuzzo (S) 2010 (31)	27	14.8	16	23.3	17.8	20	8.2%	0.22 [-0.44, 0.88]	2010
Biancuzzo (J) 2010 (31)	26.5	18	17	32	25.3	18	8.1%	-0.24 [-0.91, 0.42]	2010
Binkley 2011 (29)	15.3	13	16	22.8	15.5	16	7.8%	-0.51 [-1.22, 0.19]	2011
Heaney 2011 (25)	60	25.8	16	112.5	40.5	17	0.0%	-1.50 [-2.28, -0.71]	2011
Fisk-1 (5) 2012 (36)	4.9	10.8	8	11.9	12.5	7	0.0%	-0.57 [-1.61, 0.47]	2012
Fisk-2 (10) 2012 (36)	13.6	13.6	8	19.7	14.9	8	0.0%	-0.40 [-1.40, 0.59]	2012
Keegan 2013 (32)	24.5	9.8	8	43.3	3.7	3	0.0%	-1.95 [-3.62, -0.28]	2013
Lehman 2013 (33)	30.2	12.4	46	45.5	14.4	42	0.0%	-1.13 [-1.58, -0.68]	2013
Logan 2013 (34)	-18	12.9	13	0	12.2	23	0.0%	-1.41 [-2.18, -0.65]	2013
Nimitphong 2013 (39)	7.8	9.6	19	15.9	19.2	20	0.0%	-0.52 [-1.16, 0.12]	2013
Itkonen 2016 (35)	9.9	12.6	9	16.7	10.2	8	5.7%	-0.56 [-1.54, 0.42]	2016
Shieh 2016 (26)	30.3	17.6	19	69	32.9	19	7.6%	-1.44 [-2.16, -0.71]	2016
Tripkovic (J) 2017 (37)	14.8	17.8	67	31.7	17.7	70	11.2%	-0.95 [-1.30, -0.59]	2017
Tripkovic (B) 2017 (37)	15.8	18	66	31.1	17.6	67	11.2%	-0.85 [-1.21, -0.50]	2017
Hammami 2017 (9)	30.6	16	28	27	17	31	9.6%	0.21 [-0.30, 0.73]	2019
Nasim 2019 (27)	14.7	19	136	46.8	27.7	106	0.0%	-1.38 [-1.66, -1.10]	2019
**Total (95% CI)**			**300**			**350**	**100.0%**	**-0.56 [-0.87, -0.25]**	

Heterogeneity: Tau² = 0.19; Chi² = 35.15, df = 11 (P = 0.0002); I² = 69%
Test for overall effect: Z = 3.57 (P = 0.0004)

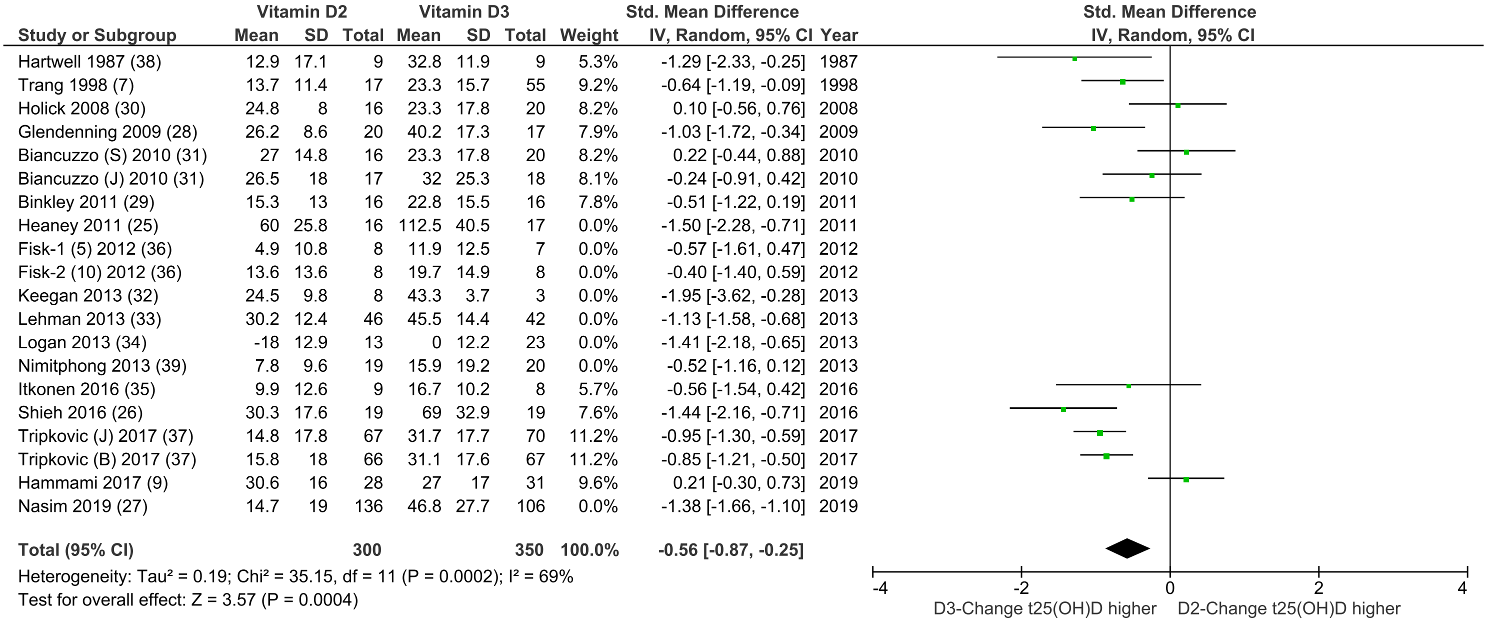

B1

Study or Subgroup	Vitamin D2 Mean	SD	Total	Vitamin D3 Mean	SD	Total	Weight	Std. Mean Difference IV, Random, 95% CI	Year
Hartwell 1987 (38)	12.9	17.1	9	32.8	11.9	9	3.8%	-1.29 [-2.33, -0.25]	1987
Trang 1998 (7)	13.7	11.4	17	23.3	15.7	55	7.1%	-0.64 [-1.19, -0.09]	1998
Holick 2008 (30)	24.8	8	16	23.3	17.8	20	6.2%	0.10 [-0.56, 0.76]	2008
Glendenning 2009 (28)	26.2	8.6	20	40.2	17.3	17	5.9%	-1.03 [-1.72, -0.34]	2009
Biancuzzo (S) 2010 (31)	27	14.8	16	23.3	17.8	20	6.2%	0.22 [-0.44, 0.88]	2010
Biancuzzo (J) 2010 (31)	26.5	18	17	32	25.3	18	6.2%	-0.24 [-0.91, 0.42]	2010
Binkley 2011 (29)	15.3	13	16	22.8	15.5	16	5.8%	-0.51 [-1.22, 0.19]	2011
Fisk-1 (5) 2012 (36)	4.9	10.8	8	11.9	12.5	7	3.8%	-0.57 [-1.61, 0.47]	2012
Fisk-2 (10) 2012 (36)	13.6	13.6	8	19.7	14.9	8	4.0%	-0.40 [-1.40, 0.59]	2012
Keegan 2013 (32)	24.5	9.8	8	43.3	3.7	3	1.9%	-1.95 [-3.62, -0.28]	2013
Lehman 2013 (33)	30.2	12.4	46	45.5	14.4	42	8.0%	-1.13 [-1.58, -0.68]	2013
Logan 2013 (34)	-18	12.9	13	0	12.2	23	5.4%	-1.41 [-2.18, -0.65]	2013
Nimitphong 2013 (39)	7.8	9.6	19	15.9	19.2	20	6.4%	-0.52 [-1.16, 0.12]	2013
Itkonen 2016 (35)	9.9	12.6	9	16.7	10.2	8	4.1%	-0.56 [-1.54, 0.42]	2016
Tripkovic (J) 2017 (37)	14.8	17.8	67	31.7	17.7	70	8.8%	-0.95 [-1.30, -0.59]	2017
Tripkovic (B) 2017 (37)	15.8	18	66	31.1	17.6	67	8.8%	-0.85 [-1.21, -0.50]	2017
Hammami 2017 (9)	30.6	16	28	27	17	31	7.4%	0.21 [-0.30, 0.73]	2019
**Total (95% CI)**			**383**			**434**	**100.0%**	**-0.62 [-0.88, -0.37]**	

Heterogeneity: Tau² = 0.16; Chi² = 41.76, df = 16 (P = 0.0004); I² = 62%
Test for overall effect: Z = 4.81 (P < 0.00001)

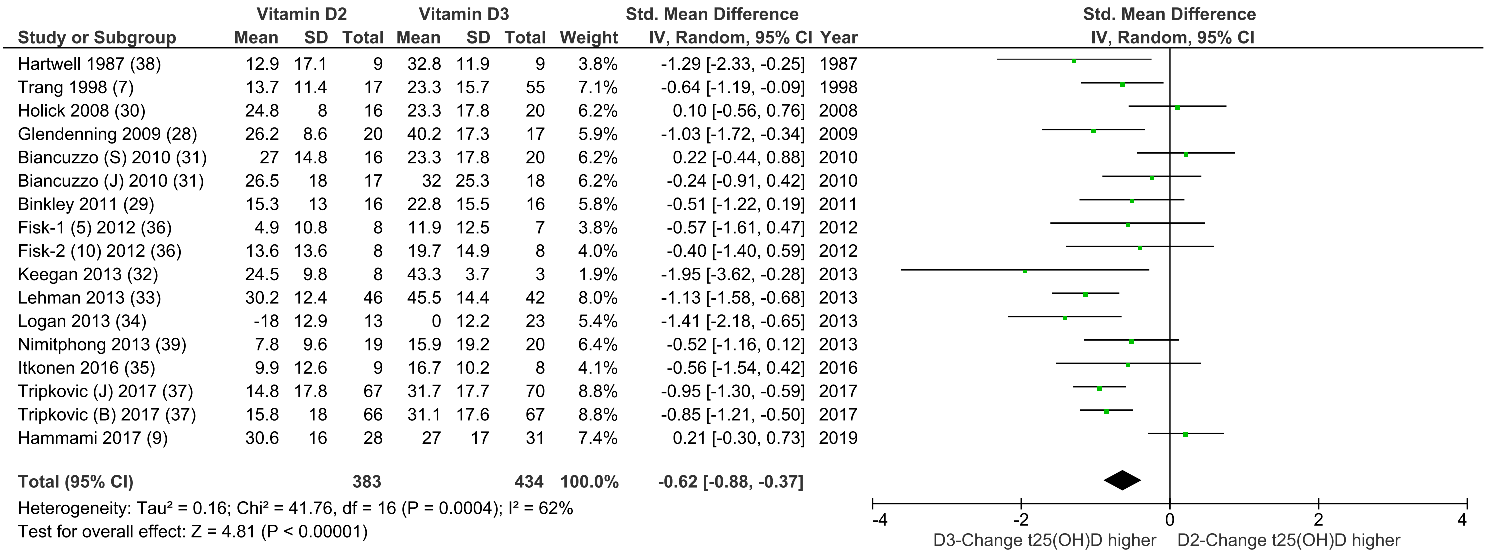

B2

Study or Subgroup	Vitamin D2 Mean	SD	Total	Vitamin D3 Mean	SD	Total	Weight	Std. Mean Difference IV, Random, 95% CI	Year
Hartwell 1987 (38)	12.9	17.1	9	32.8	11.9	9	4.9%	-1.29 [-2.33, -0.25]	1987
Trang 1998 (7)	13.7	11.4	17	23.3	15.7	55	8.4%	-0.64 [-1.19, -0.09]	1998
Holick 2008 (30)	24.8	8	16	23.3	17.8	20	7.5%	0.10 [-0.56, 0.76]	2008
Glendenning 2009 (28)	26.2	8.6	20	40.2	17.3	17	7.2%	-1.03 [-1.72, -0.34]	2009
Biancuzzo (S) 2010 (31)	27	14.8	16	23.3	17.8	20	7.5%	0.22 [-0.44, 0.88]	2010
Biancuzzo (J) 2010 (31)	26.5	18	17	32	25.3	18	7.4%	-0.24 [-0.91, 0.42]	2010
Binkley 2011 (29)	15.3	13	16	22.8	15.5	16	7.1%	-0.51 [-1.22, 0.19]	2011
Fisk-1 (5) 2012 (36)	4.9	10.8	8	11.9	12.5	7	4.9%	-0.57 [-1.61, 0.47]	2012
Fisk-2 (10) 2012 (36)	13.6	13.6	8	19.7	14.9	8	5.1%	-0.40 [-1.40, 0.59]	2012
Nimitphong 2013 (39)	7.8	9.6	19	15.9	19.2	20	7.6%	-0.52 [-1.16, 0.12]	2013
Keegan 2013 (32)	24.5	9.8	8	43.3	3.7	3	2.5%	-1.95 [-3.62, -0.28]	2013
Lehman 2013 (33)	30.2	12.4	46	45.5	14.4	42	9.2%	-1.13 [-1.58, -0.68]	2013
Logan 2013 (34)	-18	12.9	13	0	12.2	23	6.7%	-1.41 [-2.18, -0.65]	2013
Itkonen 2016 (35)	9.9	12.6	9	16.7	10.2	8	5.2%	-0.56 [-1.54, 0.42]	2016
Hammami 2017 (9)	30.6	16	28	27	17	31	8.7%	0.21 [-0.30, 0.73]	2019
**Total (95% CI)**			**250**			**297**	**100.0%**	**-0.57 [-0.87, -0.27]**	

Heterogeneity: $Tau^2 = 0.20$; $Chi^2 = 36.32$, df = 14 (P = 0.0009); $I^2 = 61\%$

Test for overall effect: Z = 3.69 (P = 0.0002)

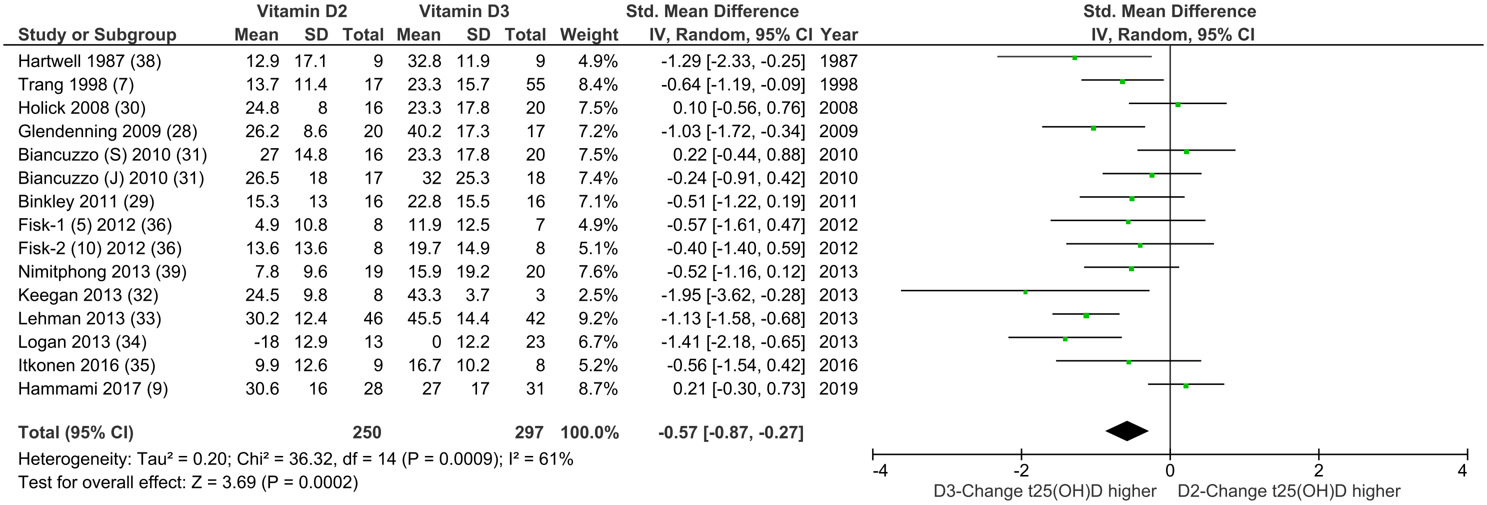

B3

Study or Subgroup	Vitamin D2 Mean	SD	Total	Vitamin D3 Mean	SD	Total	Weight	Std. Mean Difference IV, Random, 95% CI	Year
Hartwell 1987 (38)	12.9	17.1	9	32.8	11.9	9	5.5%	-1.29 [-2.33, -0.25]	1987
Trang 1998 (7)	13.7	11.4	17	23.3	15.7	55	10.0%	-0.64 [-1.19, -0.09]	1998
Holick 2008 (30)	24.8	8	16	23.3	17.8	20	8.8%	0.10 [-0.56, 0.76]	2008
Glendenning 2009 (28)	26.2	8.6	20	40.2	17.3	17	8.5%	-1.03 [-1.72, -0.34]	2009
Biancuzzo (S) 2010 (31)	27	14.8	16	23.3	17.8	20	8.8%	0.22 [-0.44, 0.88]	2010
Biancuzzo (J) 2010 (31)	26.5	18	17	32	25.3	18	8.8%	-0.24 [-0.91, 0.42]	2010
Binkley 2011 (29)	15.3	13	16	22.8	15.5	16	8.3%	-0.51 [-1.22, 0.19]	2011
Fisk-1 (5) 2012 (36)	4.9	10.8	8	11.9	12.5	7	0.0%	-0.57 [-1.61, 0.47]	2012
Fisk-2 (10) 2012 (36)	13.6	13.6	8	19.7	14.9	8	0.0%	-0.40 [-1.40, 0.59]	2012
Keegan 2013 (32)	24.5	9.8	8	43.3	3.7	3	0.0%	-1.95 [-3.62, -0.28]	2013
Lehman 2013 (33)	30.2	12.4	46	45.5	14.4	42	0.0%	-1.13 [-1.58, -0.68]	2013
Logan 2013 (34)	-18	12.9	13	0	12.2	23	0.0%	-1.41 [-2.18, -0.65]	2013
Nimitphong 2013 (39)	7.8	9.6	19	15.9	19.2	20	0.0%	-0.52 [-1.16, 0.12]	2013
Itkonen 2016 (35)	9.9	12.6	9	16.7	10.2	8	6.0%	-0.56 [-1.54, 0.42]	2016
Tripkovic (J) 2017 (37)	14.8	17.8	67	31.7	17.7	70	12.4%	-0.95 [-1.30, -0.59]	2017
Tripkovic (B) 2017 (37)	15.8	18	66	31.1	17.6	67	12.4%	-0.85 [-1.21, -0.50]	2017
Hammami 2017 (9)	30.6	16	28	27	17	31	10.5%	0.21 [-0.30, 0.73]	2019
**Total (95% CI)**			**281**			**331**	**100.0%**	**-0.49 [-0.80, -0.18]**	

Heterogeneity: Tau² = 0.17; Chi² = 29.86, df = 10 (P = 0.0009); I² = 67%

Test for overall effect: Z = 3.13 (P = 0.002)

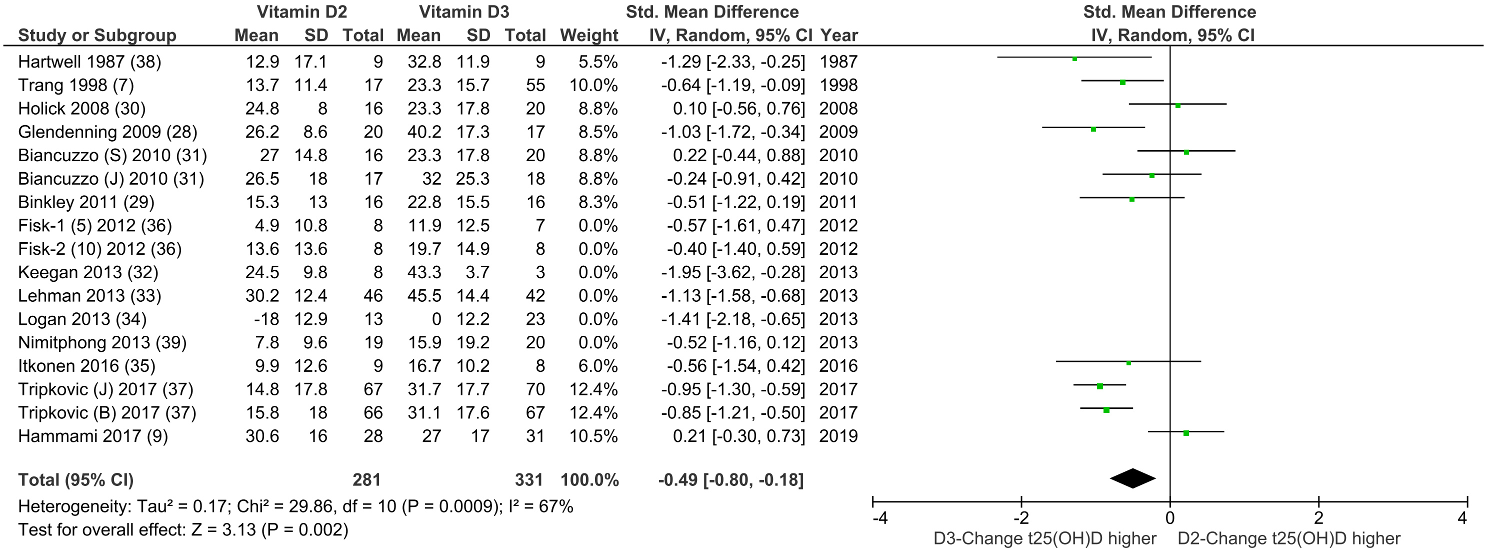

C1

Study or Subgroup	Vitamin D2 Mean	SD	Total	Vitamin D3 Mean	SD	Total	Weight	Std. Mean Difference IV, Random, 95% CI	Year
Holick 2008 (30)	24.8	8	16	23.3	17.8	20	12.3%	0.10 [-0.56, 0.76]	2008
Glendenning 2009 (28)	20.5	16.9	34	36.7	24.1	36	16.2%	-0.77 [-1.25, -0.28]	2009
Binkley 2011 (29)	15.3	13	16	22.8	15.5	16	11.3%	-0.51 [-1.22, 0.19]	2011
Fisk-2 (10) 2012 (36)	13.6	13.6	8	19.7	14.9	8	7.3%	-0.40 [-1.40, 0.59]	2012
Logan 2013 (34)	-22	16.1	25	0	12.9	30	13.4%	-1.50 [-2.11, -0.90]	2013
Tripkovic (J) 2017 (37)	14.8	17.8	67	31.7	17.7	70	19.8%	-0.95 [-1.30, -0.59]	2017
Tripkovic (B) 2017 (37)	15.8	18	66	31.1	17.6	67	19.8%	-0.85 [-1.21, -0.50]	2017
**Total (95% CI)**			**232**			**247**	**100.0%**	**-0.76 [-1.07, -0.44]**	

Heterogeneity: $Tau^2 = 0.10$; $Chi^2 = 14.41$, df = 6 (P = 0.03); $I^2 = 58\%$
Test for overall effect: Z = 4.71 (P < 0.00001)

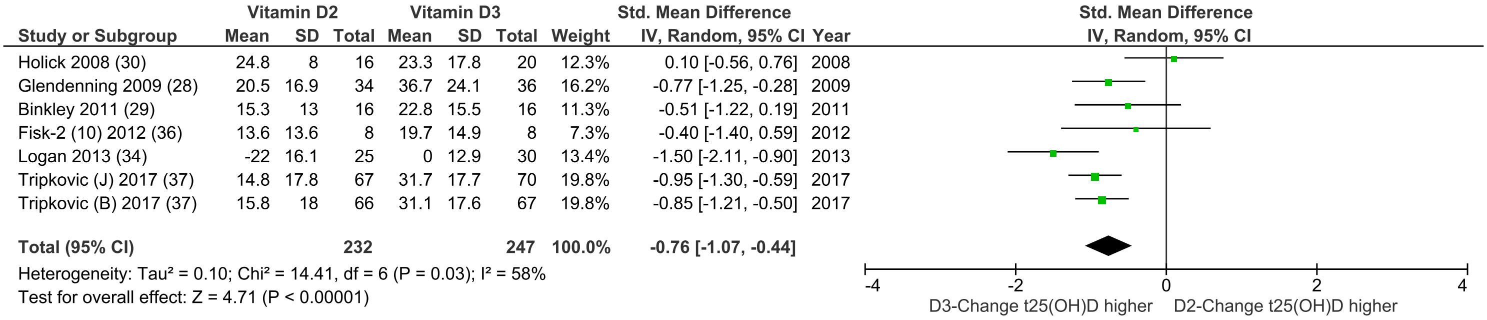

C2

Study or Subgroup	Vitamin D2<br>Mean [nMol/L]	SD [nMol/L]	Total	Vitamin D3<br>Mean [nMol/L]	SD [nMol/L]	Total	Weight	Mean Difference<br>IV, Random, 95% CI	Year
Holick 2008 (30)	24.8	8	16	23.3	17.8	20	8.7%	1.50 [-7.23, 10.23]	2008
Biancuzzo (J) 2010 (31)	26.5	18	17	32	25.3	18	5.3%	-5.50 [-19.99, 8.99]	2010
Biancuzzo (S) 2010 (31)	27	14.8	16	23.3	17.8	20	7.4%	3.70 [-6.95, 14.35]	2010
Fisk-2 (10) 2012 (36)	13.6	13.6	8	19.7	14.9	8	5.5%	-6.10 [-20.08, 7.88]	2012
Fisk-1 (5) 2012 (36)	4.9	10.8	8	11.9	12.5	7	6.6%	-7.00 [-18.91, 4.91]	2012
Logan 2013 (34)	-18	12.9	13	0	12.2	23	8.8%	-18.00 [-26.60, -9.40]	2013
Nimitphong 2013 (39)	7.8	9.6	19	15.9	19.2	20	8.2%	-8.10 [-17.56, 1.36]	2013
Keegan 2013 (32)	24.5	9.8	8	43.3	3.7	3	9.3%	-18.80 [-26.78, -10.82]	2013
Lehman 2013 (33)	30.2	12.4	46	45.5	14.4	42	11.1%	-15.30 [-20.94, -9.66]	2013
Itkonen 2016 (35)	9.9	12.6	9	16.7	10.2	8	7.3%	-6.80 [-17.65, 4.05]	2016
Tripkovic (J) 2017 (37)	14.8	17.8	67	31.7	17.7	70	10.9%	-16.90 [-22.85, -10.95]	2017
Tripkovic (B) 2017 (37)	15.8	18	66	31.1	17.6	67	10.8%	-15.30 [-21.35, -9.25]	2017
**Total (95% CI)**			**293**			**306**	**100.0%**	**-10.39 [-14.62, -6.16]**	

Heterogeneity: Tau² = 33.51; Chi² = 30.92, df = 11 (P = 0.001); I² = 64%

Test for overall effect: Z = 4.81 (P < 0.00001)

Mean Difference
IV, Random, 95% CI

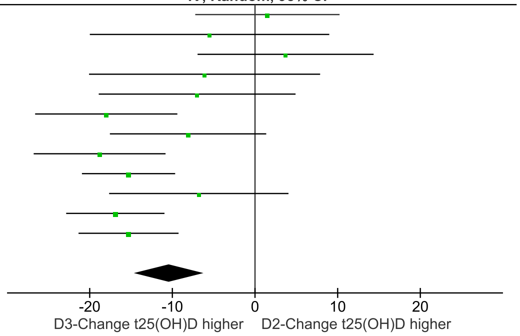

D1

Study or Subgroup	25(OH)D2 due to D2 Mean	SD	Total	25(OH)D3 due to D3 Mean	SD	Total	Weight	Std. Mean Difference IV, Random, 95% CI	Year
Glendenning 2009 (28)	28.9	23.9	20	36	10.6	17	10.9%	-0.37 [-1.02, 0.29]	2009
Fisk-1 (5) 2012 (36)	9.2	5.3	8	12	12.5	7	5.7%	-0.28 [-1.30, 0.74]	2012
Fisk-2 (10) 2012 (36)	17.6	12	8	19.8	15	8	6.1%	-0.15 [-1.14, 0.83]	2012
Keegan 2013 (32)	29.5	8.9	8	45.3	1.6	3	2.5%	-1.83 [-3.47, -0.20]	2013
Lehman 2013 (33)	50	18	46	46.7	21	42	17.4%	0.17 [-0.25, 0.59]	2013
Nimitphong 2013 (39)	22	9.2	19	16.2	18.8	20	11.3%	0.38 [-0.25, 1.01]	2013
Itkonen 2016 (35)	31.3	11	9	18.5	10.7	8	5.5%	1.12 [0.07, 2.16]	2016
Tripkovic (B) 2017 (37)	30	30.5	66	35.5	24.7	67	20.2%	-0.20 [-0.54, 0.14]	2017
Tripkovic (J) 2017 (37)	28.6	70.9	67	34.5	24	70	20.4%	-0.11 [-0.45, 0.22]	2017
**Total (95% CI)**			**251**			**242**	**100.0%**	**-0.04 [-0.31, 0.23]**	

Heterogeneity: Tau² = 0.06; Chi² = 14.16, df = 8 (P = 0.08); I² = 44%
Test for overall effect: Z = 0.29 (P = 0.77)

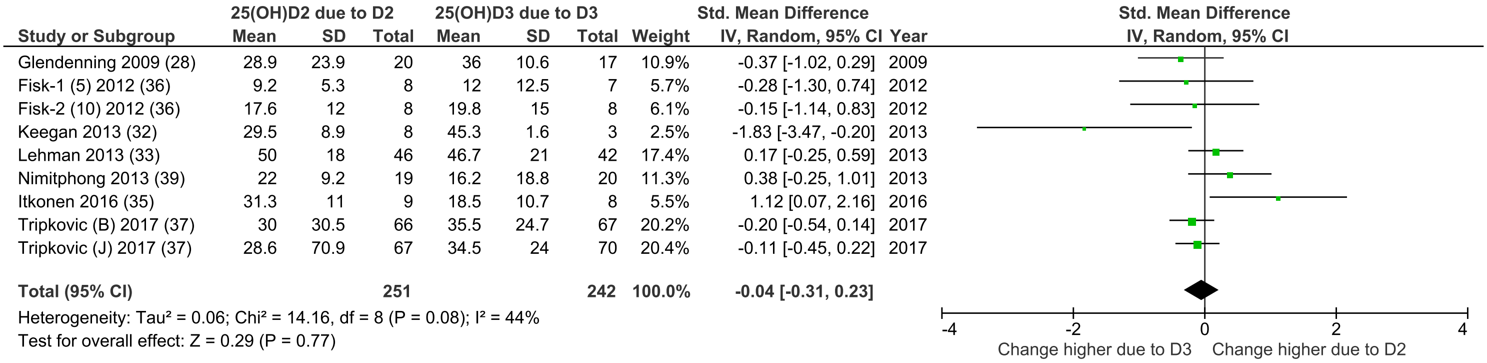

D2

Study or Subgroup	25(OH)D2 due to D2 Mean	SD	Total	25(OH)D3 due to D3 Mean	SD	Total	Weight	Std. Mean Difference IV, Random, 95% CI	Year
Glendenning 2009 (28)	28.9	23.9	20	36	10.6	17	18.0%	-0.37 [-1.02, 0.29]	2009
Fisk-1 (5) 2012 (36)	9.2	5.3	8	12	12.5	7	11.4%	-0.28 [-1.30, 0.74]	2012
Fisk-2 (10) 2012 (36)	17.6	12	8	19.8	15	8	11.9%	-0.15 [-1.14, 0.83]	2012
Keegan 2013 (32)	29.5	8.9	8	45.3	1.6	3	5.8%	-1.83 [-3.47, -0.20]	2013
Lehman 2013 (33)	50	18	46	46.7	21	42	23.6%	0.17 [-0.25, 0.59]	2013
Nimitphong 2013 (39)	22	9.2	19	16.2	18.8	20	18.4%	0.38 [-0.25, 1.01]	2013
Itkonen 2016 (35)	31.3	11	9	18.5	10.7	8	11.0%	1.12 [0.07, 2.16]	2016
**Total (95% CI)**			**118**			**105**	**100.0%**	**0.01 [-0.42, 0.45]**	

Heterogeneity: $Tau^2 = 0.16$; $Chi^2 = 12.57$, df = 6 (P = 0.05); $I^2 = 52\%$
Test for overall effect: Z = 0.05 (P = 0.96)

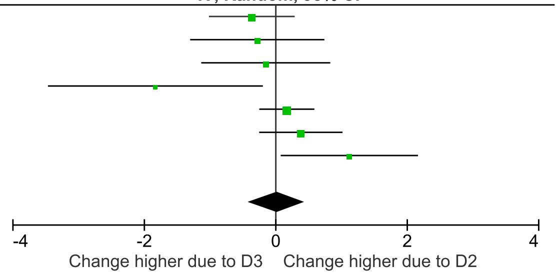

D3

	25(OH)D2 due to D2			25(OH)D3 due to D3				Std. Mean Difference		Std. Mean Difference
**Study or Subgroup**	**Mean**	**SD**	**Total**	**Mean**	**SD**	**Total**	**Weight**	**IV, Random, 95% CI**	**Year**	**IV, Random, 95% CI**
Glendenning 2009 (28)	17.7	24.1	34	28.8	14.3	36	19.1%	-0.56 [-1.04, -0.08]	2009	
Fisk-2 (10) 2012 (36)	17.6	12	8	19.8	15	8	4.5%	-0.15 [-1.14, 0.83]	2012	
Tripkovic (J) 2017 (37)	28.6	70.9	67	34.5	24	70	38.8%	-0.11 [-0.45, 0.22]	2017	
Tripkovic (B) 2017 (37)	30	30.5	66	35.5	24.8	67	37.6%	-0.20 [-0.54, 0.14]	2017	
**Total (95% CI)**			**175**			**181**	**100.0%**	**-0.23 [-0.44, -0.02]**		

Heterogeneity: Tau² = 0.00; Chi² = 2.34, df = 3 (P = 0.50); I² = 0%

Test for overall effect: Z = 2.17 (P = 0.03)

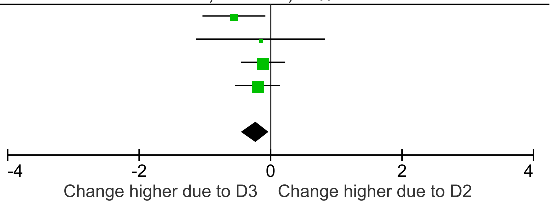

D4

Study or Subgroup	25(OH)D2 due to D2 Mean	SD	Total	25(OH)D3 due to D3 Mean	SD	Total	Weight	Std. Mean Difference IV, Random, 95% CI	Year
Glendenning 2009 (28)	28.9	23.9	20	36	10.6	17	19.0%	-0.37 [-1.02, 0.29]	2009
Fisk-1 (5) 2012 (36)	9.2	5.3	8	12	12.5	7	0.0%	-0.28 [-1.30, 0.74]	2012
Fisk-2 (10) 2012 (36)	17.6	12	8	19.8	15	8	0.0%	-0.15 [-1.14, 0.83]	2012
Keegan 2013 (32)	29.5	8.9	8	45.3	1.6	3	0.0%	-1.83 [-3.47, -0.20]	2013
Lehman 2013 (33)	50	18	46	46.7	21	42	0.0%	0.17 [-0.25, 0.59]	2013
Nimitphong 2013 (39)	22	9.2	19	16.2	18.8	20	0.0%	0.38 [-0.25, 1.01]	2013
Itkonen 2016 (35)	31.3	11	9	18.5	10.7	8	9.5%	1.12 [0.07, 2.16]	2016
Tripkovic (B) 2017 (37)	30	30.5	66	35.5	24.7	67	35.5%	-0.20 [-0.54, 0.14]	2017
Tripkovic (J) 2017 (37)	28.6	70.9	67	34.5	24	70	35.9%	-0.11 [-0.45, 0.22]	2017
**Total (95% CI)**			**162**			**162**	**100.0%**	**-0.07 [-0.43, 0.28]**	

Heterogeneity: Tau² = 0.06; Chi² = 6.14, df = 3 (P = 0.10); I² = 51%
Test for overall effect: Z = 0.40 (P = 0.69)

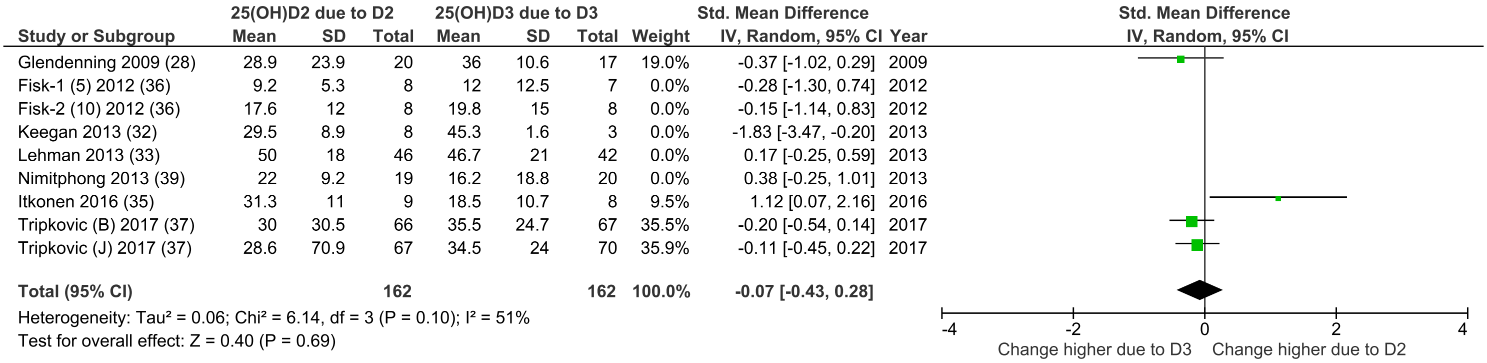

Supplementary Figures S5 Subgroup analyses on net changes in serum 25(OH)D concentrations. In the figures, “vitamin D2” and “vitamin D3” denotes the change in serum 25(OH)D concentrations from baseline (net change) in the D2 and D3 group respectively, and “Total” denotes the cumulative number of all included comparisons. IV, inverse variance; t25(OH)D, total 25(OH)D concentration. Trials are sorted out by type of subgroup: A. >60% vs. ≤60% subjects with baseline 25(OH)D <50 nmol/L. Excluding low quality studies (33,34,36,39), the SMD between D2 and D3 in predominantly deficient subjects was -0.22 (95% CI -0.62, 0.18; I2=61%; p=0.29). B. >50% vs. ≤50% Caucasian subjects. Excluding the low quality studies (34,36,39), the SMD between D2 and D3 in predominantly Caucasian subjects was -0.86 (95% CI -1.09, -0.64; I2=0%; p<0.00001) vs -0.08 (-0.42, 0.27); 39%; p=0.67) in the other subgroup (p=0.0002). C. < 65 vs. ≥65 years of age. Excluding the low quality studies (32–34,36,39), the SMD between D2 and D3 in subjects younger than 65 was -0.65 (95% CI -1.05, -0.25; I2=69%; p=0.001) vs -0.28 (-0.72, 0.16; 52%; p=0.21) in the older subgroup (p difference=0.23). D. >70% vs. ≤70% female subjects. Excluding the low quality studies (32–34,36,39), the SMD between D2 and D3 in predominantly female subjects was -0.90 (95% CI -1.14, -0.66; I2=0%; p<0.00001) vs -0.14 (-0.45, 0.18; 36%; p=0.4) in the other subgroup (p=0.0001); E. Latitude ≥45^0 N, 30-45^0 N vs. <30^0 N at which the study was conducted. Excluding the low quality studies (32–34,36,39), the SMD between D2 and D3 at a latitude of ≥45^0 N was -0.90 (95% CI -1.14, -0.66; I2=0%; p<0.00001), between 30-45^0 N was -0.23 (-0.57, 0.11; 27%; p=0.18), and at a latitude of <30^0 N was -0.38 (-1.60, 0.83; 88%; p=0.54); F. Dose ≤25 vs. >25 µg. Excluding the low quality studies (32–34,36,39), the SMD between D2 and D3 when dosage was ≤25 mcg/d was -0.51 (95% CI -0.89, -0.14; I2=67%; p=0.007), vs -0.47 (-1.04, 0.11; 67%; p=0.11) at a dose >25 mcg/d (p=0.89); G. Calcium present in the supplement, yes vs.

no. Excluding the low quality studies (32–34,36,39), the SMD between D2 and D3, where calcium was also present in the treatment was -1.11 (95% CI -1.68, -0.53; I2=0%; p=0.0002) vs -0.39 (-0.72, -0.05; 69%; p=0.02) in the other subgroup (p=0.03).

A

Study or Subgroup	Vitamin D2 Mean	Vitamin D2 SD	Vitamin D2 Total	Vitamin D3 Mean	Vitamin D3 SD	Vitamin D3 Total	Weight	Std. Mean Difference IV, Random, 95% CI	Year
**1.5.1 Baseline of >60% subjects <50 nmol/l 25(OH)D**									
Trang 1998 (7)	13.7	11.4	17	23.3	15.7	55	9.7%	-0.64 [-1.19, -0.09]	1998
Holick 2008 (30)	24.8	8	16	23.3	17.8	20	8.8%	0.10 [-0.56, 0.76]	2008
Glendenning 2009 (28)	26.2	8.6	20	40.2	17.3	17	8.4%	-1.03 [-1.72, -0.34]	2009
Biancuzzo (J) 2010 (31)	26.5	18	17	32	25.3	18	8.7%	-0.24 [-0.91, 0.42]	2010
Biancuzzo (S) 2010 (31)	27	14.8	16	23.3	17.8	20	8.7%	0.22 [-0.44, 0.88]	2010
Fisk-2 (10) 2012 (36)	13.6	13.6	8	19.7	14.9	8	6.1%	-0.40 [-1.40, 0.59]	2012
Fisk-1 (5) 2012 (36)	4.9	10.8	8	11.9	12.5	7	5.7%	-0.57 [-1.61, 0.47]	2012
Lehman 2013 (33)	30.2	12.4	46	45.5	14.4	42	10.7%	-1.13 [-1.58, -0.68]	2013
Hammami 2017 (9)	30.6	16	28	27	17	31	10.1%	0.21 [-0.30, 0.73]	2019
**Subtotal (95% CI)**			**176**			**218**	**77.1%**	**-0.39 [-0.77, -0.00]**	
Heterogeneity: Tau² = 0.23; Chi² = 25.40, df = 8 (P = 0.001); I² = 68%									
Test for overall effect: Z = 1.97 (P = 0.05)									
**1.5.2 Baseline of =< 60% subjects <50 nmol/l 25(OH)D**									
Logan 2013 (34)	-18	12.9	13	0	12.2	23	7.8%	-1.41 [-2.18, -0.65]	2013
Nimitphong 2013 (39)	7.8	9.6	19	15.9	19.2	20	8.9%	-0.52 [-1.16, 0.12]	2013
Itkonen 2016 (35)	9.9	12.6	9	16.7	10.2	8	6.2%	-0.56 [-1.54, 0.42]	2016
**Subtotal (95% CI)**			**41**			**51**	**22.9%**	**-0.83 [-1.42, -0.24]**	
Heterogeneity: Tau² = 0.11; Chi² = 3.45, df = 2 (P = 0.18); I² = 42%									
Test for overall effect: Z = 2.77 (P = 0.006)									
**Total (95% CI)**			**217**			**269**	**100.0%**	**-0.49 [-0.82, -0.16]**	
Heterogeneity: Tau² = 0.21; Chi² = 31.42, df = 11 (P = 0.0009); I² = 65%									
Test for overall effect: Z = 2.91 (P = 0.004)									
Test for subgroup differences: Chi² = 1.53, df = 1 (P = 0.22), I² = 34.7%									

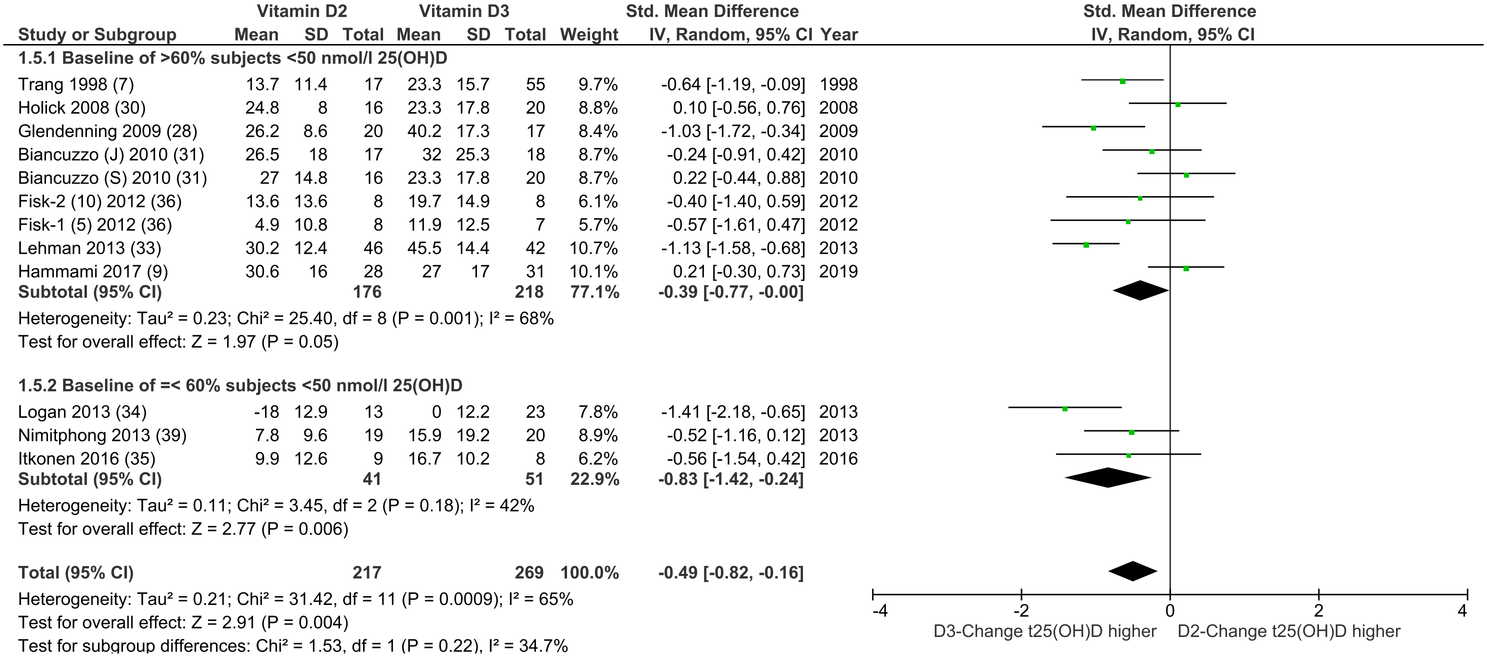

B

Study or Subgroup	Vitamin D2 Mean	SD	Total	Vitamin D3 Mean	SD	Total	Weight	Std. Mean Difference IV, Random, 95% CI	Year
**4.9.1 >50% Caucasian**									
Hartwell 1987 (38)	12.9	17.1	9	32.8	11.9	9	4.5%	-1.29 [-2.33, -0.25]	1987
Binkley 2011 (29)	15.3	13	16	22.8	15.5	16	6.9%	-0.51 [-1.22, 0.19]	2011
Fisk-2 (10) 2012 (36)	13.6	13.6	8	19.7	14.9	8	4.7%	-0.40 [-1.40, 0.59]	2012
Fisk-1 (5) 2012 (36)	4.9	10.8	8	11.9	12.5	7	4.5%	-0.57 [-1.61, 0.47]	2012
Logan 2013 (34)	-18	12.9	13	0	12.2	23	6.4%	-1.41 [-2.18, -0.65]	2013
Itkonen 2016 (35)	9.9	12.6	9	16.7	10.2	8	4.8%	-0.56 [-1.54, 0.42]	2016
Tripkovic (J) 2017 (37)	14.8	17.8	67	31.7	17.7	70	10.6%	-0.95 [-1.30, -0.59]	2017
Tripkovic (B) 2017 (37)	15.8	18	66	31.1	17.6	67	10.6%	-0.85 [-1.21, -0.50]	2017
**Subtotal (95% CI)**			**196**			**208**	**53.1%**	**-0.87 [-1.08, -0.66]**	
Heterogeneity: Tau² = 0.00; Chi² = 5.31, df = 7 (P = 0.62); I² = 0%									
Test for overall effect: Z = 8.28 (P < 0.00001)									
**4.9.2 =<50% Caucasian**									
Trang 1998 (7)	13.7	11.4	17	23.3	15.7	55	8.4%	-0.64 [-1.19, -0.09]	1998
Holick 2008 (30)	24.8	8	16	23.3	17.8	20	7.4%	0.10 [-0.56, 0.76]	2008
Biancuzzo (J) 2010 (31)	26.5	18	17	32	25.3	18	7.3%	-0.24 [-0.91, 0.42]	2010
Biancuzzo (S) 2010 (31)	27	14.8	16	23.3	17.8	20	7.4%	0.22 [-0.44, 0.88]	2010
Nimitphong 2013 (39)	7.8	9.6	19	15.9	19.2	20	7.5%	-0.52 [-1.16, 0.12]	2013
Hammami 2017 (9)	30.6	16	28	27	17	31	8.9%	0.21 [-0.30, 0.73]	2019
**Subtotal (95% CI)**			**113**			**164**	**46.9%**	**-0.15 [-0.46, 0.17]**	
Heterogeneity: Tau² = 0.06; Chi² = 8.07, df = 5 (P = 0.15); I² = 38%									
Test for overall effect: Z = 0.90 (P = 0.37)									
**Total (95% CI)**			**309**			**372**	**100.0%**	**-0.51 [-0.79, -0.24]**	
Heterogeneity: Tau² = 0.15; Chi² = 33.00, df = 13 (P = 0.002); I² = 61%									
Test for overall effect: Z = 3.72 (P = 0.0002)									
Test for subgroup differences: Chi² = 14.19, df = 1 (P = 0.0002), I² = 93.0%									

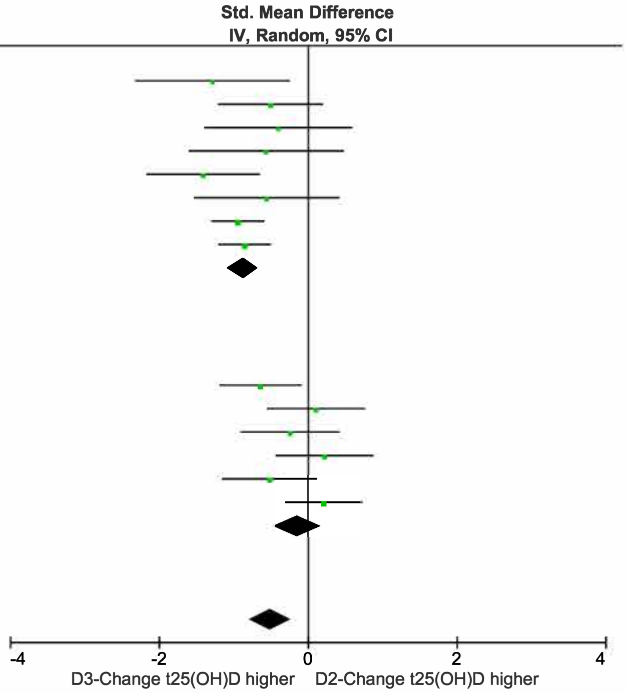

C

Study or Subgroup	Vitamin D2 Mean	Vitamin D2 SD	Vitamin D2 Total	Vitamin D3 Mean	Vitamin D3 SD	Vitamin D3 Total	Weight	Std. Mean Difference IV, Random, 95% CI	Year
**4.7.1 Age < 65y**									
Hartwell 1987 (38)	12.9	17.1	9	32.8	11.9	9	3.8%	-1.29 [-2.33, -0.25]	1987
Trang 1998 (7)	13.7	11.4	17	23.3	15.7	55	7.1%	-0.64 [-1.19, -0.09]	1998
Fisk-2 (10) 2012 (36)	13.6	13.6	8	19.7	14.9	8	4.0%	-0.40 [-1.40, 0.59]	2012
Fisk-1 (5) 2012 (36)	4.9	10.8	8	11.9	12.5	7	3.8%	-0.57 [-1.61, 0.47]	2012
Keegan 2013 (32)	24.5	9.8	8	43.3	3.7	3	1.9%	-1.95 [-3.62, -0.28]	2013
Lehman 2013 (33)	30.2	12.4	46	45.5	14.4	42	8.0%	-1.13 [-1.58, -0.68]	2013
Nimitphong 2013 (39)	7.8	9.6	19	15.9	19.2	20	6.4%	-0.52 [-1.16, 0.12]	2013
Logan 2013 (34)	-18	12.9	13	0	12.2	23	5.4%	-1.41 [-2.18, -0.65]	2013
Itkonen 2016 (35)	9.9	12.6	9	16.7	10.2	8	4.1%	-0.56 [-1.54, 0.42]	2016
Tripkovic (J) 2017 (37)	14.8	17.8	67	31.7	17.7	70	8.8%	-0.95 [-1.30, -0.59]	2017
Tripkovic (B) 2017 (37)	15.8	18	66	31.1	17.6	67	8.8%	-0.85 [-1.21, -0.50]	2017
Hammami 2017 (9)	30.6	16	28	27	17	31	7.4%	0.21 [-0.30, 0.73]	2019
**Subtotal (95% CI)**			**298**			**343**	**69.6%**	**-0.77 [-1.05, -0.49]**	
Heterogeneity: Tau² = 0.12; Chi² = 25.07, df = 11 (P = 0.009); I² = 56%									
Test for overall effect: Z = 5.38 (P < 0.00001)									
**4.7.2 Age >= 65y**									
Holick 2008 (30)	24.8	8	16	23.3	17.8	20	6.2%	0.10 [-0.56, 0.76]	2008
Glendenning 2009 (28)	26.2	8.6	20	40.2	17.3	17	5.9%	-1.03 [-1.72, -0.34]	2009
Biancuzzo (J) 2010 (31)	26.5	18	17	32	25.3	18	6.2%	-0.24 [-0.91, 0.42]	2010
Biancuzzo (S) 2010 (31)	27	14.8	16	23.3	17.8	20	6.2%	0.22 [-0.44, 0.88]	2010
Binkley 2011 (29)	15.3	13	16	22.8	15.5	16	5.8%	-0.51 [-1.22, 0.19]	2011
**Subtotal (95% CI)**			**85**			**91**	**30.4%**	**-0.28 [-0.72, 0.16]**	
Heterogeneity: Tau² = 0.13; Chi² = 8.42, df = 4 (P = 0.08); I² = 52%									
Test for overall effect: Z = 1.26 (P = 0.21)									
**Total (95% CI)**			**383**			**434**	**100.0%**	**-0.62 [-0.88, -0.37]**	
Heterogeneity: Tau² = 0.16; Chi² = 41.76, df = 16 (P = 0.0004); I² = 62%									
Test for overall effect: Z = 4.81 (P < 0.00001)									
Test for subgroup differences: Chi² = 3.32, df = 1 (P = 0.07), I² = 69.9%									

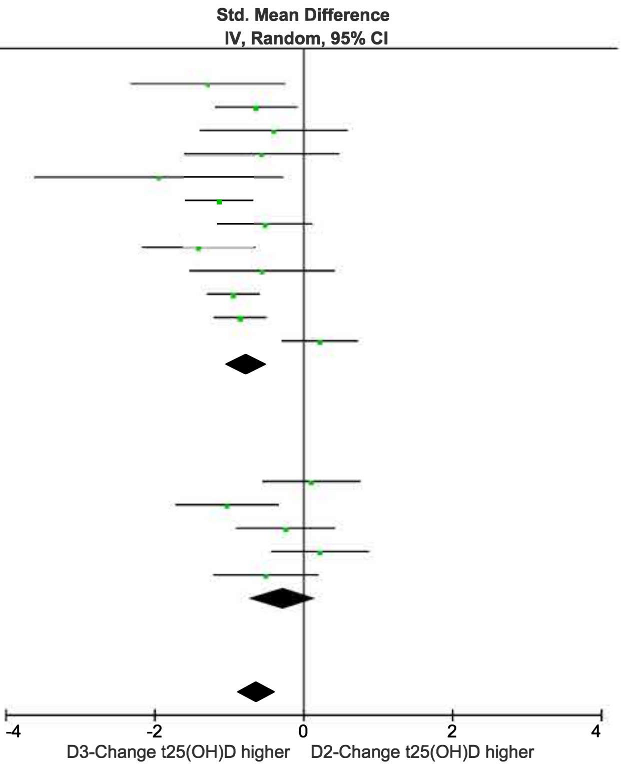

D

Study or Subgroup	Vitamin D2 Mean	SD	Total	Vitamin D3 Mean	SD	Total	Weight	Std. Mean Difference IV, Random, 95% CI	Year
**4.8.1 >70% women**									
Hartwell 1987 (38)	12.9	17.1	9	32.8	11.9	9	4.1%	-1.29 [-2.33, -0.25]	1987
Keegan 2013 (32)	24.5	9.8	8	43.3	3.7	3	2.1%	-1.95 [-3.62, -0.28]	2013
Nimitphong 2013 (39)	7.8	9.6	19	15.9	19.2	20	6.8%	-0.52 [-1.16, 0.12]	2013
Logan 2013 (34)	-18	12.9	13	0	12.2	23	5.8%	-1.41 [-2.18, -0.65]	2013
Itkonen 2016 (35)	9.9	12.6	9	16.7	10.2	8	4.4%	-0.56 [-1.54, 0.42]	2016
Tripkovic (J) 2017 (37)	14.8	17.8	67	31.7	17.7	70	9.3%	-0.95 [-1.30, -0.59]	2017
Tripkovic (B) 2017 (37)	15.8	18	66	31.1	17.6	67	9.3%	-0.85 [-1.21, -0.50]	2017
**Subtotal (95% CI)**			**191**			**200**	**41.8%**	**-0.92 [-1.13, -0.70]**	
Heterogeneity: Tau² = 0.00; Chi² = 5.73, df = 6 (P = 0.45); I² = 0%									
Test for overall effect: Z = 8.49 (P < 0.00001)									
**4.8.2 =< 70% women**									
Trang 1998 (7)	13.7	11.4	17	23.3	15.7	55	7.5%	-0.64 [-1.19, -0.09]	1998
Holick 2008 (30)	24.8	8	16	23.3	17.8	20	6.6%	0.10 [-0.56, 0.76]	2008
Biancuzzo (J) 2010 (31)	26.5	18	17	32	25.3	18	6.6%	-0.24 [-0.91, 0.42]	2010
Biancuzzo (S) 2010 (31)	27	14.8	16	23.3	17.8	20	6.6%	0.22 [-0.44, 0.88]	2010
Binkley 2011 (29)	15.3	13	16	22.8	15.5	16	6.2%	-0.51 [-1.22, 0.19]	2011
Fisk-2 (10) 2012 (36)	13.6	13.6	8	19.7	14.9	8	4.4%	-0.40 [-1.40, 0.59]	2012
Fisk-1 (5) 2012 (36)	4.9	10.8	8	11.9	12.5	7	4.1%	-0.57 [-1.61, 0.47]	2012
Lehman 2013 (33)	30.2	12.4	46	45.5	14.4	42	8.4%	-1.13 [-1.58, -0.68]	2013
Hammami 2017 (9)	30.6	16	28	27	17	31	7.9%	0.21 [-0.30, 0.73]	2019
**Subtotal (95% CI)**			**172**			**217**	**58.2%**	**-0.33 [-0.70, 0.03]**	
Heterogeneity: Tau² = 0.19; Chi² = 22.32, df = 8 (P = 0.004); I² = 64%									
Test for overall effect: Z = 1.79 (P = 0.07)									
**Total (95% CI)**			**363**			**417**	**100.0%**	**-0.60 [-0.86, -0.33]**	
Heterogeneity: Tau² = 0.16; Chi² = 40.62, df = 15 (P = 0.0004); I² = 63%									
Test for overall effect: Z = 4.40 (P < 0.0001)									
Test for subgroup differences: Chi² = 7.39, df = 1 (P = 0.007), I² = 86.5%									

Std. Mean Difference
IV, Random, 95% CI

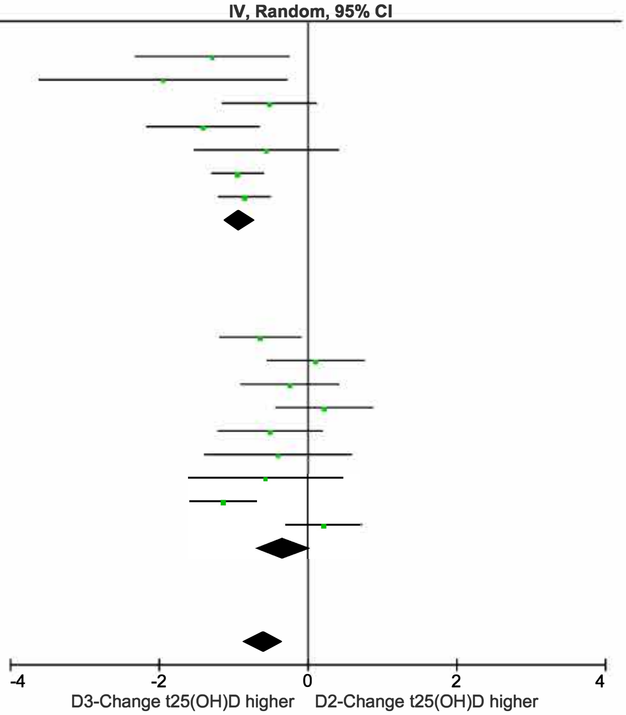

E

Study or Subgroup	Vitamin D2 Mean	SD	Total	Vitamin D3 Mean	SD	Total	Weight	Std. Mean Difference IV, Random, 95% CI	Year
**4.14.1 >=45 degrees North**									
Hartwell 1987 (38)	12.9	17.1	9	32.8	11.9	9	3.8%	-1.29 [-2.33, -0.25]	1987
Fisk-1 (5) 2012 (36)	4.9	10.8	8	11.9	12.5	7	3.8%	-0.57 [-1.61, 0.47]	2012
Fisk-2 (10) 2012 (36)	13.6	13.6	8	19.7	14.9	8	4.0%	-0.40 [-1.40, 0.59]	2012
Lehman 2013 (33)	30.2	12.4	46	45.5	14.4	42	8.0%	-1.13 [-1.58, -0.68]	2013
Itkonen 2016 (35)	9.9	12.6	9	16.7	10.2	8	4.1%	-0.56 [-1.54, 0.42]	2016
Tripkovic (J) 2017 (37)	14.8	17.8	67	31.7	17.7	70	8.8%	-0.95 [-1.30, -0.59]	2017
Tripkovic (B) 2017 (37)	15.8	18	66	31.1	17.6	67	8.8%	-0.85 [-1.21, -0.50]	2017
**Subtotal (95% CI)**			**213**			**211**	**41.5%**	**-0.91 [-1.12, -0.71]**	
Heterogeneity: Tau² = 0.00; Chi² = 3.48, df = 6 (P = 0.75); I² = 0%									
Test for overall effect: Z = 8.90 (P < 0.00001)									
**4.14.2 30-<45 degrees North**									
Trang 1998 (7)	13.7	11.4	17	23.3	15.7	55	7.1%	-0.64 [-1.19, -0.09]	1998
Holick 2008 (30)	24.8	8	16	23.3	17.8	20	6.2%	0.10 [-0.56, 0.76]	2008
Biancuzzo (S) 2010 (31)	27	14.8	16	23.3	17.8	20	6.2%	0.22 [-0.44, 0.88]	2010
Biancuzzo (J) 2010 (31)	26.5	18	17	32	25.3	18	6.2%	-0.24 [-0.91, 0.42]	2010
Binkley 2011 (29)	15.3	13	16	22.8	15.5	16	5.8%	-0.51 [-1.22, 0.19]	2011
Keegan 2013 (32)	24.5	9.8	8	43.3	3.7	3	1.9%	-1.95 [-3.62, -0.28]	2013
**Subtotal (95% CI)**			**90**			**132**	**33.4%**	**-0.31 [-0.71, 0.09]**	
Heterogeneity: Tau² = 0.11; Chi² = 9.37, df = 5 (P = 0.10); I² = 47%									
Test for overall effect: Z = 1.52 (P = 0.13)									
**4.14.3 <30 degrees North**									
Glendenning 2009 (28)	26.2	8.6	20	40.2	17.3	17	5.9%	-1.03 [-1.72, -0.34]	2009
Logan 2013 (34)	-18	12.9	13	0	12.2	23	5.4%	-1.41 [-2.18, -0.65]	2013
Nimitphong 2013 (39)	7.8	9.6	19	15.9	19.2	20	6.4%	-0.52 [-1.16, 0.12]	2013
Hammami 2017 (9)	30.6	16	28	27	17	31	7.4%	0.21 [-0.30, 0.73]	2019
**Subtotal (95% CI)**			**80**			**91**	**25.2%**	**-0.65 [-1.38, 0.07]**	
Heterogeneity: Tau² = 0.44; Chi² = 15.23, df = 3 (P = 0.002); I² = 80%									
Test for overall effect: Z = 1.76 (P = 0.08)									
**Total (95% CI)**			**383**			**434**	**100.0%**	**-0.62 [-0.88, -0.37]**	
Heterogeneity: Tau² = 0.16; Chi² = 41.76, df = 16 (P = 0.0004); I² = 62%									
Test for overall effect: Z = 4.81 (P < 0.00001)									
Test for subgroup differences: Chi² = 7.02, df = 2 (P = 0.03), I² = 71.5%									

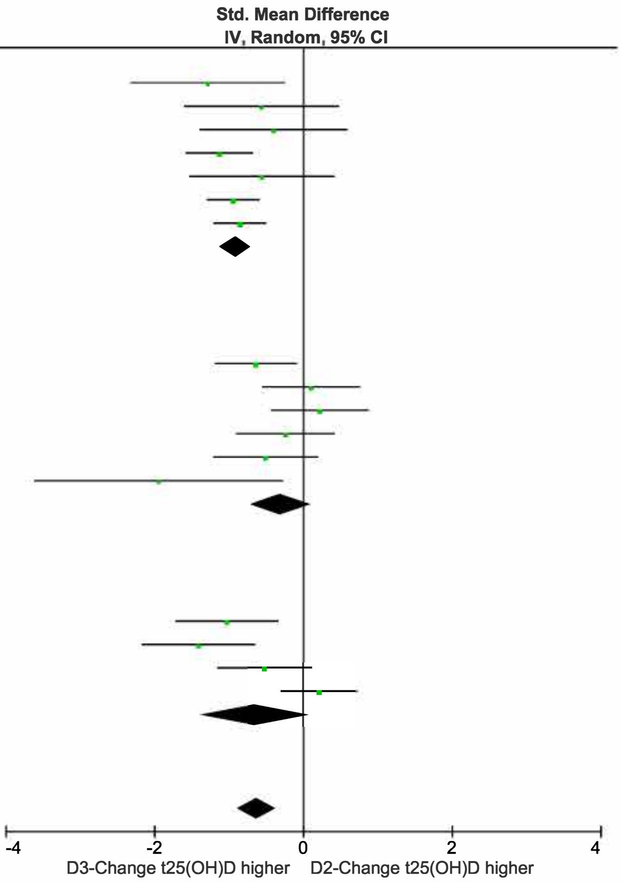

F

Study or Subgroup	Vitamin D2 Mean	Vitamin D2 SD	Vitamin D2 Total	Vitamin D3 Mean	Vitamin D3 SD	Vitamin D3 Total	Weight	Std. Mean Difference IV, Random, 95% CI	Year
**4.16.1 Dose =< 25 mcg/d**									
Holick 2008 (30)	24.8	8	16	23.3	17.8	20	6.2%	0.10 [-0.56, 0.76]	2008
Glendenning 2009 (28)	26.2	8.6	20	40.2	17.3	17	5.9%	-1.03 [-1.72, -0.34]	2009
Biancuzzo (J) 2010 (31)	26.5	18	17	32	25.3	18	6.2%	-0.24 [-0.91, 0.42]	2010
Biancuzzo (S) 2010 (31)	27	14.8	16	23.3	17.8	20	6.2%	0.22 [-0.44, 0.88]	2010
Fisk-1 (5) 2012 (36)	4.9	10.8	8	11.9	12.5	7	3.8%	-0.57 [-1.61, 0.47]	2012
Fisk-2 (10) 2012 (36)	13.6	13.6	8	19.7	14.9	8	4.0%	-0.40 [-1.40, 0.59]	2012
Logan 2013 (34)	-18	12.9	13	0	12.2	23	5.4%	-1.41 [-2.18, -0.65]	2013
Nimitphong 2013 (39)	7.8	9.6	19	15.9	19.2	20	6.4%	-0.52 [-1.16, 0.12]	2013
Itkonen 2016 (35)	9.9	12.6	9	16.7	10.2	8	4.1%	-0.56 [-1.54, 0.42]	2016
Tripkovic (J) 2017 (37)	14.8	17.8	67	31.7	17.7	70	8.8%	-0.95 [-1.30, -0.59]	2017
Tripkovic (B) 2017 (37)	15.8	18	66	31.1	17.6	67	8.8%	-0.85 [-1.21, -0.50]	2017
**Subtotal (95% CI)**			**259**			**278**	**66.0%**	**-0.59 [-0.88, -0.30]**	
Heterogeneity: Tau² = 0.12; Chi² = 22.49, df = 10 (P = 0.01); I² = 56%									
Test for overall effect: Z = 4.00 (P < 0.0001)									
**4.16.2 Dose >25 mcg/d**									
Hartwell 1987 (38)	12.9	17.1	9	32.8	11.9	9	3.8%	-1.29 [-2.33, -0.25]	1987
Trang 1998 (7)	13.7	11.4	17	23.3	15.7	55	7.1%	-0.64 [-1.19, -0.09]	1998
Binkley 2011 (29)	15.3	13	16	22.8	15.5	16	5.8%	-0.51 [-1.22, 0.19]	2011
Lehman 2013 (33)	30.2	12.4	46	45.5	14.4	42	8.0%	-1.13 [-1.58, -0.68]	2013
Keegan 2013 (32)	24.5	9.8	8	43.3	3.7	3	1.9%	-1.95 [-3.62, -0.28]	2013
Hammami 2017 (9)	30.6	16	28	27	17	31	7.4%	0.21 [-0.30, 0.73]	2019
**Subtotal (95% CI)**			**124**			**156**	**34.0%**	**-0.73 [-1.28, -0.18]**	
Heterogeneity: Tau² = 0.32; Chi² = 19.22, df = 5 (P = 0.002); I² = 74%									
Test for overall effect: Z = 2.61 (P = 0.009)									
**Total (95% CI)**			**383**			**434**	**100.0%**	**-0.62 [-0.88, -0.37]**	
Heterogeneity: Tau² = 0.16; Chi² = 41.76, df = 16 (P = 0.0004); I² = 62%									
Test for overall effect: Z = 4.81 (P < 0.00001)									
Test for subgroup differences: Chi² = 0.20, df = 1 (P = 0.66), I² = 0%									

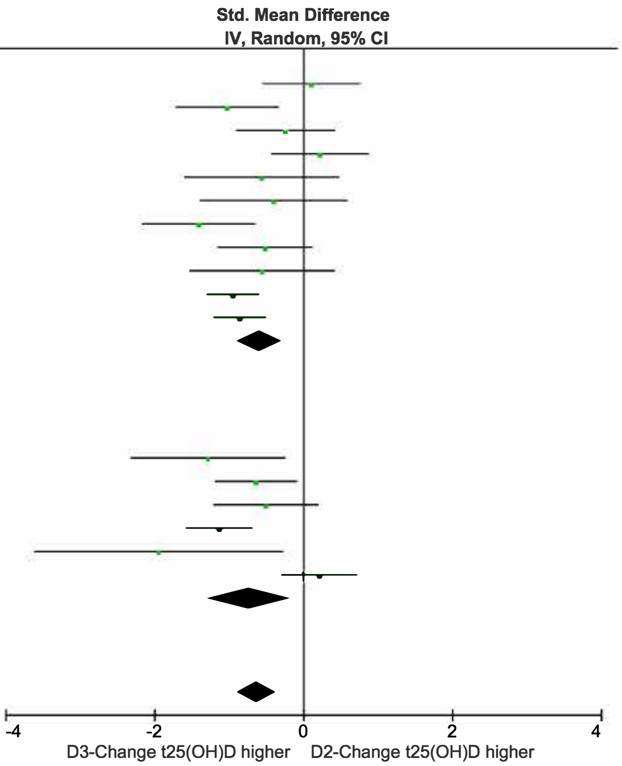

G

Study or Subgroup	Vitamin D2 Mean	Vitamin D2 SD	Vitamin D2 Total	Vitamin D3 Mean	Vitamin D3 SD	Vitamin D3 Total	Weight	Std. Mean Difference IV, Random, 95% CI	Year
**4.11.1 Calcium in treatment**									
Hartwell 1987 (38)	12.9	17.1	9	32.8	11.9	9	3.8%	-1.29 [-2.33, -0.25]	1987
Glendenning 2009 (28)	26.2	8.6	20	40.2	17.3	17	5.9%	-1.03 [-1.72, -0.34]	2009
Nimitphong 2013 (39)	7.8	9.6	19	15.9	19.2	20	6.4%	-0.52 [-1.16, 0.12]	2013
**Subtotal (95% CI)**			**48**			**46**	**16.1%**	**-0.84 [-1.27, -0.42]**	
Heterogeneity: Tau² = 0.00; Chi² = 1.97, df = 2 (P = 0.37); I² = 0%									
Test for overall effect: Z = 3.86 (P = 0.0001)									
**4.11.2 No Calcium in treatment**									
Trang 1998 (7)	13.7	11.4	17	23.3	15.7	55	7.1%	-0.64 [-1.19, -0.09]	1998
Holick 2008 (30)	24.8	8	16	23.3	17.8	20	6.2%	0.10 [-0.56, 0.76]	2008
Biancuzzo (S) 2010 (31)	27	14.8	16	23.3	17.8	20	6.2%	0.22 [-0.44, 0.88]	2010
Biancuzzo (J) 2010 (31)	26.5	18	17	32	25.3	18	6.2%	-0.24 [-0.91, 0.42]	2010
Binkley 2011 (29)	15.3	13	16	22.8	15.5	16	5.8%	-0.51 [-1.22, 0.19]	2011
Fisk-2 (10) 2012 (36)	13.6	13.6	8	19.7	14.9	8	4.0%	-0.40 [-1.40, 0.59]	2012
Fisk-1 (5) 2012 (36)	4.9	10.8	8	11.9	12.5	7	3.8%	-0.57 [-1.61, 0.47]	2012
Keegan 2013 (32)	24.5	9.8	8	43.3	3.7	3	1.9%	-1.95 [-3.62, -0.28]	2013
Lehman 2013 (33)	30.2	12.4	46	45.5	14.4	42	8.0%	-1.13 [-1.58, -0.68]	2013
Logan 2013 (34)	-18	12.9	13	0	12.2	23	5.4%	-1.41 [-2.18, -0.65]	2013
Itkonen 2016 (35)	9.9	12.6	9	16.7	10.2	8	4.1%	-0.56 [-1.54, 0.42]	2016
Tripkovic (B) 2017 (37)	15.8	18	66	31.1	17.6	67	8.8%	-0.85 [-1.21, -0.50]	2017
Tripkovic (J) 2017 (37)	14.8	17.8	67	31.7	17.7	70	8.8%	-0.95 [-1.30, -0.59]	2017
Hammami 2017 (9)	30.6	16	28	27	17	31	7.4%	0.21 [-0.30, 0.73]	2019
**Subtotal (95% CI)**			**335**			**388**	**83.9%**	**-0.57 [-0.86, -0.28]**	
Heterogeneity: Tau² = 0.18; Chi² = 39.00, df = 13 (P = 0.0002); I² = 67%									
Test for overall effect: Z = 3.84 (P = 0.0001)									
**Total (95% CI)**			**383**			**434**	**100.0%**	**-0.62 [-0.88, -0.37]**	
Heterogeneity: Tau² = 0.16; Chi² = 41.76, df = 16 (P = 0.0004); I² = 62%									
Test for overall effect: Z = 4.81 (P < 0.00001)									
Test for subgroup differences: Chi² = 1.08, df = 1 (P = 0.30), I² = 7.2%									

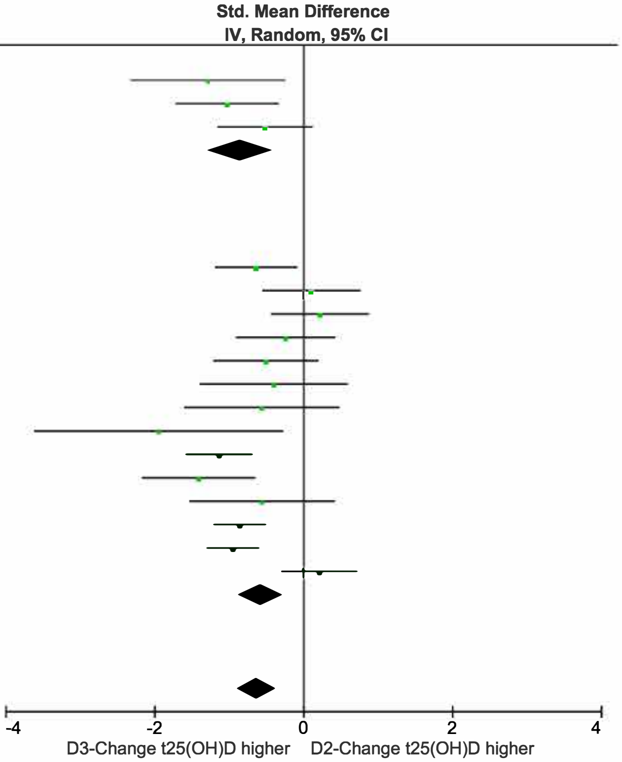

Supplement: Multimedia component 1 [file mmc1.pdf]
